# Automatic curation of LTR retrotransposon libraries from plant genomes through machine learning

**DOI:** 10.1515/jib-2021-0036

**Published:** 2022-07-12

**Authors:** Simon Orozco-Arias, Mariana S. Candamil-Cortes, Paula A. Jaimes, Estiven Valencia-Castrillon, Reinel Tabares-Soto, Gustavo Isaza, Romain Guyot

**Affiliations:** Department of Computer Science, Universidad Autónoma de Manizales, Manizales, Colombia; Department of Systems and Informatics, Universidad de Caldas, Manizales, Colombia; Department of Electronics and Automation, Universidad Autónoma de Manizales, Manizales, Colombia; Institut de Recherche pour le Développement, CIRAD, Univ. Montpellier, Montpellier, France

**Keywords:** curation, deep neural networks, *k*-mer-based methods, LTR retrotransposons, machine learning, nesting insertions

## Abstract

Transposable elements are mobile sequences that can move and insert themselves into chromosomes, activating under internal or external stimuli, giving the organism the ability to adapt to the environment. Annotating transposable elements in genomic data is currently considered a crucial task to understand key aspects of organisms such as phenotype variability, species evolution, and genome size, among others. Because of the way they replicate, LTR retrotransposons are the most common transposable elements in plants, accounting in some cases for up to 80% of all DNA information. To annotate these elements, a reference library is usually created, a curation process is performed, eliminating TE fragments and false positives and then annotated in the genome using the homology method. However, the curation process can take weeks, requires extensive manual work and the execution of multiple time-consuming bioinformatics software. Here, we propose a machine learning-based approach to perform this process automatically on plant genomes, obtaining up to 91.18% F1-score. This approach was tested with four plant species, obtaining up to 93.6% F1-score (*Oryza granulata*) in only 22.61 s, where bioinformatics methods took approximately 6 h. This acceleration demonstrates that the ML-based approach is efficient and could be used in massive sequencing projects.

## 1 Introduction

Due to the growing boom of massive sequencing projects [1], thanks to the reduction of sequencing costs, a need has been created for a new generation of tools that can process a large amount of data efficiently and automatically in short periods of time [2]. Particularly, the analysis of transposable elements that represent the vast majority of plant genomes, require a special attention, considering their impact on genome structure, dynamics and evolution [3]. Machine Learning (ML) algorithms have the ability to use this amount of data and learn from it how to execute a given task fitting parameters of a model to a specific dataset [4]. Inside ML, one of the most used models currently are based on neural networks (also called Deep Learning or DL), which uses nonparametric architectures with different types of neurons, activation functions and connections to fit complex associations between input and output data [5]. ML and also DL have been implemented to solve problems in biology and genomics [6, 7], and specially in transposable elements (TEs) [8, 9].

TEs are considered as major contributors to the genome evolution and adaptation [10], to have significant relations to gene regulation and genome plasticity and evolution [11], having important impacts on the generation of mutations, genetic polymorphisms [12], the generation of biodiversity, and during speciation events [13]. They constitute the dynamic portion of the DNA since they are mobile genetic structures, which have the ability to integrate into new positions in genomes and sometimes increase their copy number over time [14]. Also, TEs is one of the contributors to the genome size, as the same as polyploidy events, and segmental duplications [15].

Based on their transposition mechanism, TEs can be divided into Class I (or Retrotransposons), which use as an “copy and paste” mechanism using as intermediate of replication the RNA molecule, and Class II (or Transposons), which follow the “cut and paste” strategy, use as an intermediate the DNA molecule [16]. Among Class I elements, Long Terminal Repeats Retrotransposons (LTR-RTs) are the most abundant in copy number and diversity in plants [17], constituting up to 75% of nuclear DNA [18], even 80% of angiosperms [15]. In plants, LTR-RTs are sub classified into superfamilies (Gypsy and Copia), and in several lineages/families [19].

To avoid harmful mutations, the host genome uses processes to silence or interrupt TEs life cycle [20]. These processes create many fragment or non-autonomous copies of TEs and balance the copy number of those elements inside the genome. On the other hand, the complex dynamics of TEs have shown burst of insertions in specific sections of the genome [21], producing the presence of numerous insertions of transposable elements into other TEs, with the direct consequence of inactivating the activity of the first element. Such insertions are called nested insertions [22–24]. While multiple insertions of large transposable elements can have a profound impact on the structure of the inserted element, insertion of smaller and non-coding elements can go unnoticed by structural TE detection algorithms. As a consequence, these algorithms can predict the presence of a complete element but whose sequence could be in fact nested by other type of transposable elements. The direct use of these sequences as reference without curation can lead to misidentification and misinterpretation of the TE composition in whole genome sequences. Thus, it is recommendable that sequences must be curated for use as reference TE libraries, which is a considerable work when done manually.

Within bioinformatics, there are different approaches for detecting nested elements, especially based on their structure such as TE-greedy-nester [24] and TEnest [25]. Nevertheless, these software require complex installation processes and have many dependencies such as BLAST [26], LTR_FINDER [27], GenomeTools [28] which results in long run times and possible failures linked to one of the dependencies. Also, others TE detection software, such as EDTA [29] employ a filter module, with the aim of avoid false positive discoveries but does not integrate filters to remove nested inserts.

Here, a ML-based approach was developed to curate automatically libraries of LTR-RTs in plant genomes. This model predicts in seconds (compared with conventional bioinformatics methods that take hours to complete) which sequences must be filtered in order to create a reference library, thanks to neural network performance and the utilization of graphic processing unit (GPU) [30]. The ML-based curator identifies LTR-RTs sequences that present nested insertions or their overall length does not match the one stipulated in the literature for each lineage/family. Additionally, this model can be re-trained regularly with new datasets released in massive sequencing projects, improving each time its performance. This curator can be used as additional module to existing LTR-RT predictor to filter and increase the quality of the library produced, as well as reducing the manual work done by experts and the overall execution time.

## 2 Methodology

To address the problem of curating sequences automatically, a dataset composed of sequences considered as intact and sequences with different types of nested insertions was designed. Using this dataset, common ML algorithms were trained as two types of neural networks, an FNN and a CNN. Finally, generalization and runtime tests were performed to observe the usability of the proposed model.

### 2.1 Training dataset: complete LTR-RT sequences versus nested LTR-RT

The ML models were trained in a supervised manner. Thus, it was necessary to create a labelled dataset composed of LTR-RT sequence belaying full LTR-RTs and nested inserted LTR-RTs. First, available libraries like Repbase [31], RepetDB [32] and PGSB [33] were employed. To increase the number of sequences LTR_STRUC [34] and EDTA [29] were used in additional genomes that were not present in the databases above mentioned. Next, a script was developed in order to apply the same filters used in InpactorDB [35]. Sequences that successfully passed all InpactorDB’s filters were kept as putative complete and intact elements and labelled as zero. Elements that were eliminated in each of the filters were taken as nested insertions of TEs in different ways and a label (one to four) was given. These labels constituted four possible types of inserted sequences: 1) a nested LTR-RT element into the predicted LTR-RT element with domains belonging to different superfamily between them (i.e. Gypsy versus Copia), 2) a nested LTR-RT element into the predicted LTR-RT elements with domains belonging to different lineages/familly, 3) predicted LTR-RT elements showing a length increase compared to the in the literature [17] with a tolerance of 20% and 4) predicted LTR-RT elements showing Class II insertions. Then, we followed two workflows: the first one, using the sequences and all the labels, a multiclass problem was defined with five classes, where zero are putative complete sequences and from one to four are the different types of insertions. This dataset was called “five-label dataset”. The second one consisted in join nested insertions types (labels from one to four) in one new label named class one. This dataset was called “two-label dataset”. In this way the problem became to binary classification between non-nested versus nested elements.

To evaluate the dataset obtained, a Principal Component Analysis (PCA) and t-distributed stochastic neighbour embedding (t-SNE) techniques was used, plotting in a figure the two main components of the datasets.

### 2.2 Feature extraction and pre-processing

The first step in designing and implementing a classifier based on machine learning is to perform a feature extraction process. The goal is to obtain numerical data that represent as informatively as possible the samples contained in the training set. Due to the categorical nature of genomic data, this activity is crucial to be able to use ML models [36]. *K*-mers frequencies were used as features using 1 ≤ *k* ≤ 6 due to this approach seems to be useful for machine learning algorithms [37]. To this converted data set, scaling and dimension reduction techniques were applied using principal component analysis (PCA) with an explained variance of 96% (reduction of the initial number of features from 5460 to 2254). The resulting dataset was used to train conventional ML models (not based on neural networks) and for experiments with the fully connected neural network architecture.

Although *k*-mers frequencies have proven to be very useful for training LTR retrotransposon classifiers, this form of representation loses relevant information such as the positions of the original nucleotides. For this reason, it was decided to employ another form of representation, but this time in two dimensions, through the one-hot coding scheme [38]. In this case, for each sequence a 5-row matrix is constructed, equivalent to each of the nucleotides and the unidentified “N” with *m* number of columns (length of the longest sequence in the dataset). Thus, for *n* sequences, a matrix with a dimensionality of 5 × *m* × *n* is obtained. This form of representation was used to train a convolutional neural network, with the objective of employing positional information of the LTR retrotransposon sequences.

We took into account the F1-score metric, considering that the dataset is unbalanced, the F1-score metric was applied and taken into account, since such metric contributes significantly to the knowledge of the behaviour and generalization of the used model [36]. Finally, accuracy, precision and recall of the models with higher performance were calculated. It should be noted that the data partition used was 80% for training, 10% for validation (validation dataset) and 10% for testing (test dataset). All experiments were performed using Python 3.7 with the Scikit-Learn library version 0.24.0 and TensorFlow version 2.2.0.

### 2.3 Experiments using ML models

For the first experimental analysis, different ML algorithms [39] were used, including KNN (K-nearest neighbors), SVM (support vector machine), linear models such as LR (logistic regression), LDA (linear discriminant analysis), NB (naive Bayesian classifier), MLP (multi-layer perceptron) and models based on decision trees (DT) such as RF (random forest). The main difference with the linear and non-linear ML-classifiers is that the results do not vary when having features at different scales. For this purpose, parameters were adjusted, considering accuracy and F1-score performance. Table 1 shows the variation ranges established for each of the model parameters.

**Table 1:** Value ranges for the parameters based on [36].

Algorithm	Parameter	Step	Range	Description
KNN	n_neighbors	1	1–100	Number of neighbors
SVC	gamma = 1 × 10^−6^ C	10	10–100	Regularization parameter
LR	C	0.1	0.1–1	Inverse regularization strength
LDA	tol	0.0001	0.0001–0.001	Absolute threshold for a singular value of X to
	tol			be considered significant
NB	var_smoothing	1 × 10^−2^	1 × 10^−1^–1 × 10^−19^	Portion of the largest variance of all features
				that is added to variances for calculation stability
MLP	Solver = ’lbfgs’, alpha			
	= 0.5, hidden_layer_sizes	50	50–500	Number of neurons in the hidden layers
DT	max_depth	1–10	1	The maximum depth of the tree
RF	n_estimators	10–100	10	The number of trees in the forest

### 2.4 Experiments using FNN

**Figure 1:**
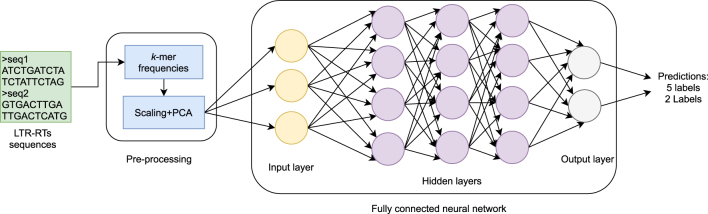
FNN architecture based on Nakano et al. [40].

A hyper-parameter tuning process was carried out taking as initial model the fully connected network published by Nakano [40]. First, different numbers of layers and different quantity of neurons per layer were tested. Also, some values of dropouts and Batch normalization were considered ranging from zero to one. Then, different activation functions such as ReLu, sigmoid and hyperbolic tangent were tested. Finally, the model was trained using the loss functions Categorical Cross entropy and Binary Cross entropy. The combination that obtained the best performance was used for the rest of the experiments (Figure 1). For the training process we used 200 epochs and a batch size of 128.

### 2.5 Experiments based on CNN

**Figure 2:**
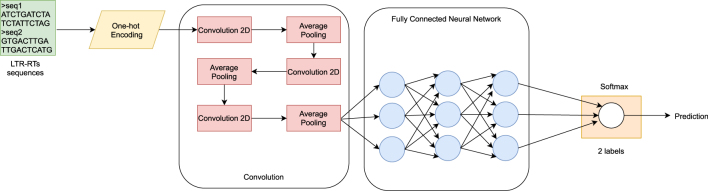
Implementation of the proposed CNN based on TERL [41].

In order to increase the F1-score percentage initially obtained, experiments were performed using the TERL convolutional network as a basis [9]. By identifying in the previous steps, a confusion between the class “0” (intact elements) with the classes “2” (Superfamily filter), “3” (lineage filter) and “4” (TE class II filter), these experiments are performed for the two-label dataset. Considering the computational expense required to use the complete dataset transformed into 2D representation, a random extraction of 30,000 samples, 15,000 of class “0” (intact elements) and 15,000 of class “1” (the union of all filters) is applied. Three convolutional layers were used with filters of 64, 32 and 32 respectively, varying the size of the kernel to obtain the best performance. A Spatial Dropout of 0.2, a Batch Normalization and an average pooling were incorporated, changing the values, to reduce the dimension, without any loss of information, necessary for the classification. Once this stage of feature extraction was completed, the data obtained is entered into the FNN with the features described above, but now considering a dropout of 0.2, since this is the one that provides the best performance in this case (Figure 2).

### 2.6 Generalization tests

Once the computational model with the highest F1-Score and accuracy was defined, generalization tests were performed with plant species with different genome sizes such as: *Coffea eugenioides* (GCA_003713205.1, 678 Mb), *Coffea humblotiana* (407 Mb) [41], *Oryza indica* (GCA_011764405.2, 355 Mb) [42] and *Oryza granulata* (GCA_003991445.1, 752 Mb) [43]. Thus, LTR_STRUC [34] and LTR_FINDER [27] software were first run to predict LTR-RTs for each genomes. Two workflows were performed, the first using conventional bioinformatics methods with filters proposed in InpactorDB [35], and the second one using the computational architecture with the highest F1-score and accuracy, identifying the accuracy percentages and execution time in each case.

### 2.7 Hardware specifications

All the analyses in this project were performed using the HPC clusters of the French Bioinformatics Institute (https://www.france-bioinformatique.fr) and IRD (https://bioinfo.ird.fr/). For the DL experiments, the Google Collaboratory platform [44] was used, which has a NVIDIA T4 GPU unit and a RAM of 16 GB. Also, a workstation with processor AMD Ryzen 3970X, GPU Nvidia RTX 2080 super and 128 GB in ram was employed.

## 3 Results

### 3.1 Descriptive analysis of the dataset

**Figure 3:**
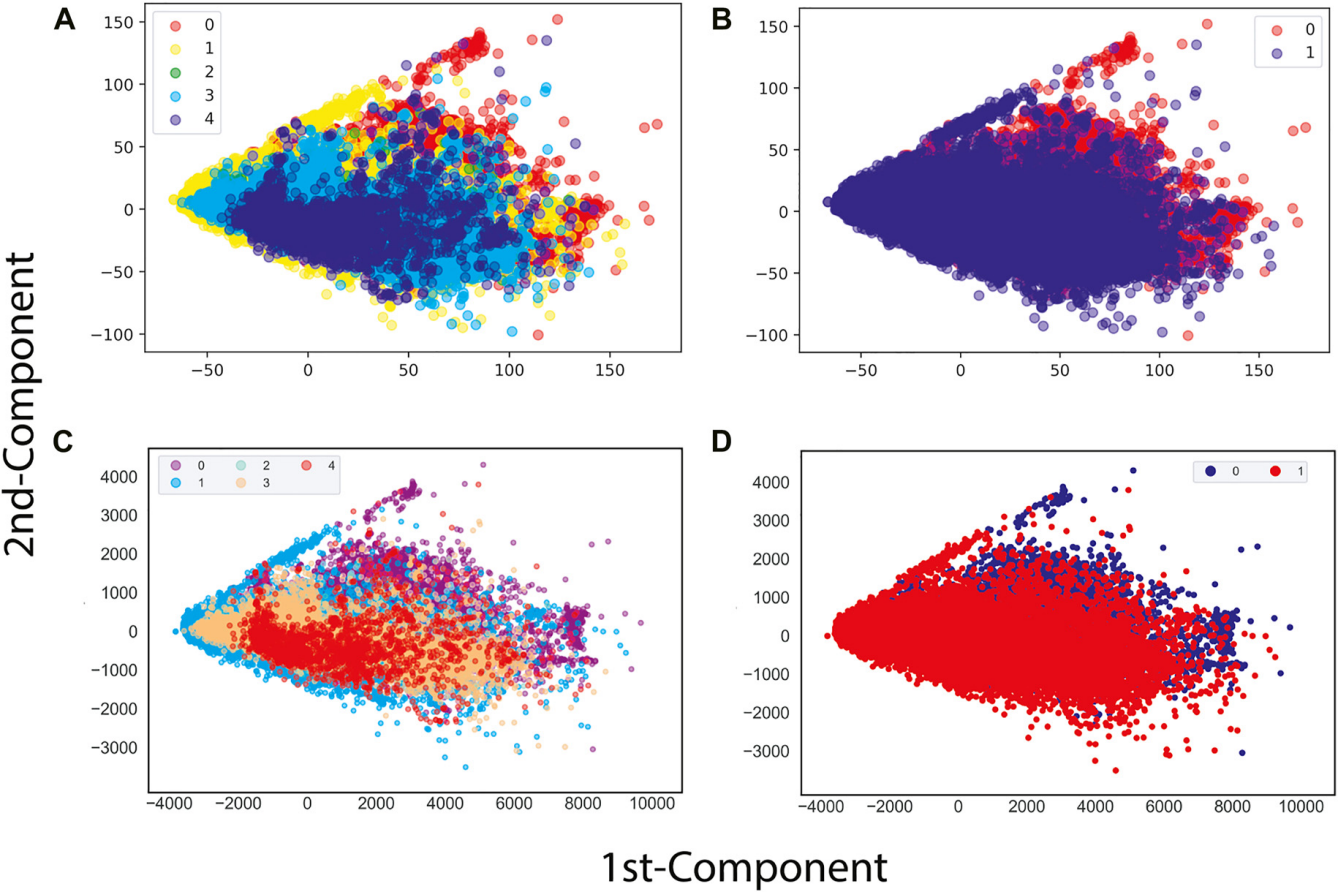
Graph of the two main components of the LTR-RT dataset: (A) PCA with five labels 0: curated sequences, 1: elements with domains belonging to two different superfamilies, 2: elements presenting domains from two or more lineages, 3: elements with lengths different from those reported in the literature, 4: elements that have TE Class II insertions, (B) PCA with 2 labels 0: curated sequences and 1: the union of classes 1, 2, 3 and 4, (C) t-SNE with the specifications of (A) and (D) t-SNE with the specifications of (B).

Once the filters were applied on the dataset, we obtained 56,442 sequences for class 0 (curated sequences) 33,874 for class 1 (elements with domains belonging to two different superfamilies), 4734 for class 2 (elements presenting domains from two or more lineages), 8568 for class 3 (elements with lengths different from those reported in the literature) and 2039 for class 4 (elements that have TE Class II insertions). Then a graphical representation of the two main components was made (Figure 3), in which an overlapping of the labels is identified. The results obtained suggest that it might be necessary to use more complex computational methods to achieve a good performance and generalization of the information.

### 3.2 A machine learning-based curator of nested inserted LTR retrotransposons in plant genomes

**Figure 4:**
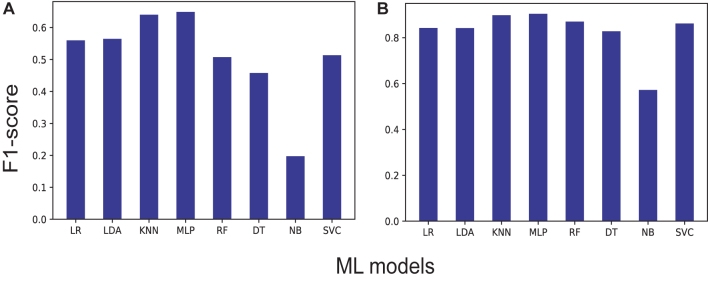
F1-score performance of the ML models used (A) using the five-label dataset and (B) using the two-label dataset. The following algorithms were used: KNN (K-nearest neighbors), SVM (support vector machine), LR (logistic regression), LDA (linear discriminant analysis), NB (naive Bayesian classifier), MLP (multi-layer perceptron), decision trees (DT), and RF (random forest).

**ML models**. Experiments were run varying the computational model, evaluating the F1-score metric with the five-label (classes 0, 1, 2, 3, 4) and two-label dataset (class 0 and the union of classes 1, 2, 3 and 4; Figure 4), in which a higher percentage is identified in the MLP and KNN models. With the five-label dataset, the performance does not exceed 65% and with the two-label dataset the performance reaches a maximum of 90% (Supplementary Material S1).

**Figure 5:**
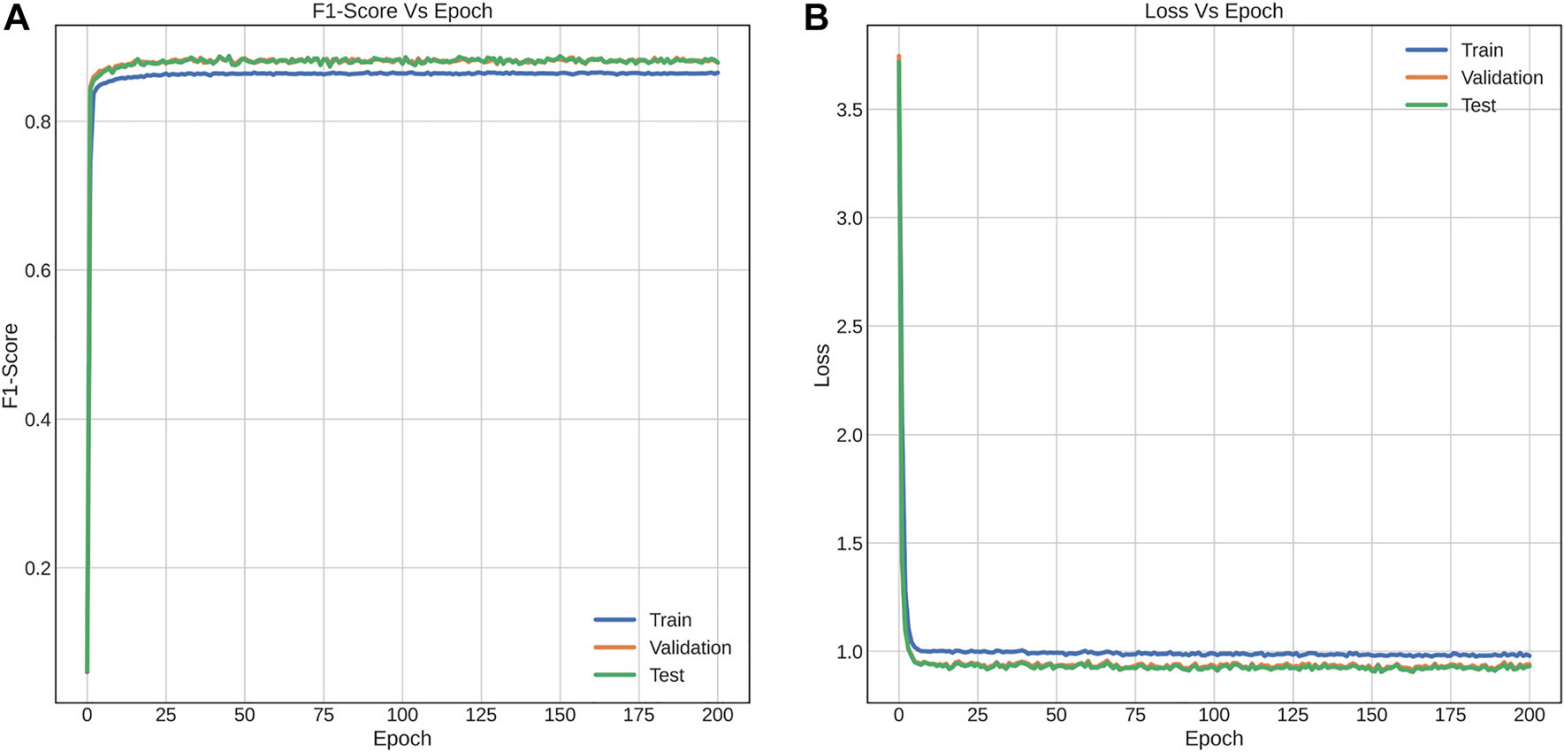
Training curves with FNN implemented for multiclass classification (A) and F1-score versus epochs (B) Loss versus epochs.

**Figure 6:**
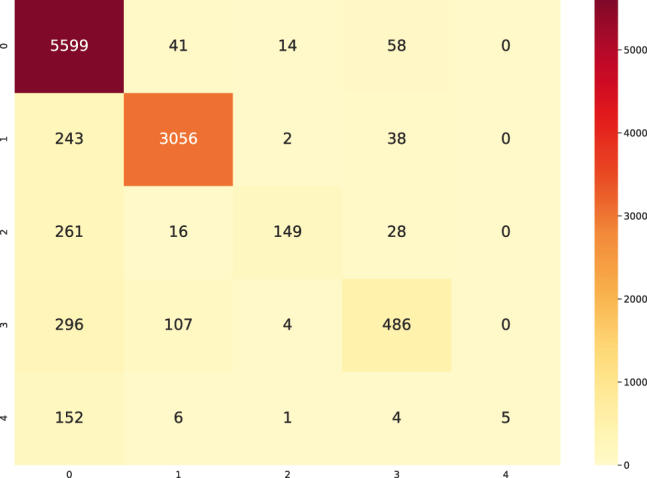
Confusion matrix made with the test data produced by the FNN with multiclass classification. 0, 1, 2, 3 and 4 refer to the classes previously defined.

**Performance with FNN.** For each the dataset used, experiments were performed using NN based on the FNN proposed in [40] (Figure 1) using dropout of 0.5 and Batch Normalization of 0.99 in the hidden layers. Figure 5 shows the training curves for the five-label dataset, which presents a performance of 88.75% (test dataset) for the F1-score metric and Figure 6 shows the confusion matrix obtained for this same dataset, with the test data (Supplementary Material S2).

**Figure 7:**
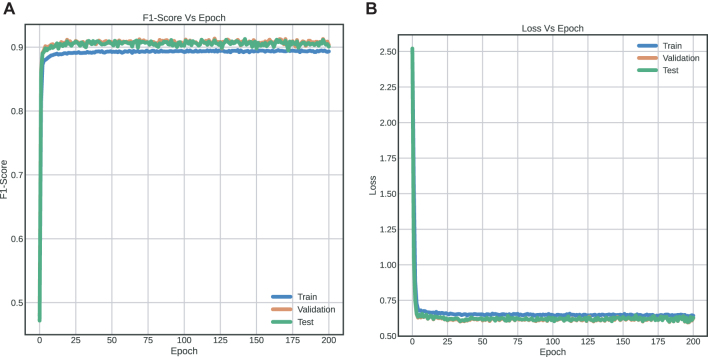
Training curves with FNN implemented for binary classification (two-label dataset) (A) F1-score vs epochs (B) Loss vs epochs.

**Figure 8:**
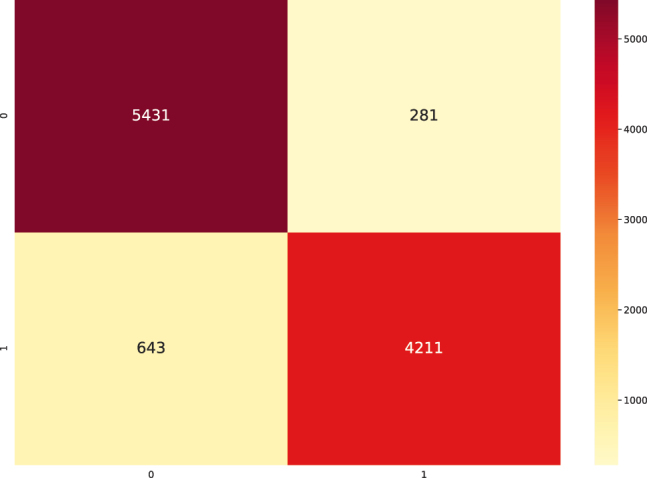
Confusion matrix performed with the test dataset produced by the FNN with binary classification (two-label dataset).

**Figure 9:**
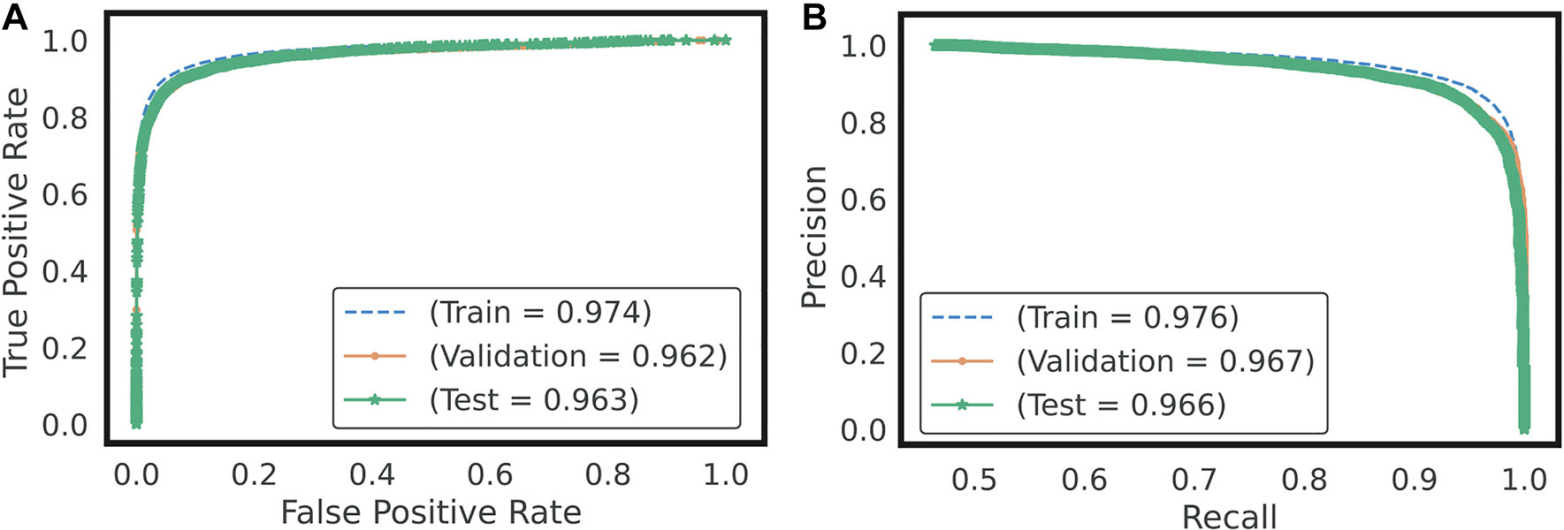
ROC curves and Precision-recall for the test data set. (A) ROC and (B) PRC curves for the test dataset.

The results indicate a confusion of class "0" (intact sequences) with classes “2”, “3” and “4”, which correspond to predicted LTR-RT elements that show domains from different superfamilies (Gypsy and Copia), predicted LTR-RT elements with domains from two or more lineages/families, predicted LTR-RT elements that shows lengths significantly different from those reported for each lineage/family in the literature, and predicted LTR-RT elements showing nested transposon insertions, respectively (Supplementary Material S3). Figure 7 and Table 2, using two-class dataset (intact elements vs all filters), show the training curve with a performance of 91.18% (in the test dataset, a 10% of the whole data) and Figure 8 shows the confusion matrix obtained for dataset. The Receiver Operating Characteristic curves (ROC) and Precision-Recall Curve (PRC) identified in Figure 9 were also plotted.

**Table 2:** Results for each metrics.

Metrics	Value
Precision	0.9140
F1-score	0.9121
Recall	0.9125
Accuracy	0.9125
Area under ROC curve (AUC)	0.963
Area under the precision recall curve (auPRC)	0.966
False positive rate	0.0355

**Figure 10:**
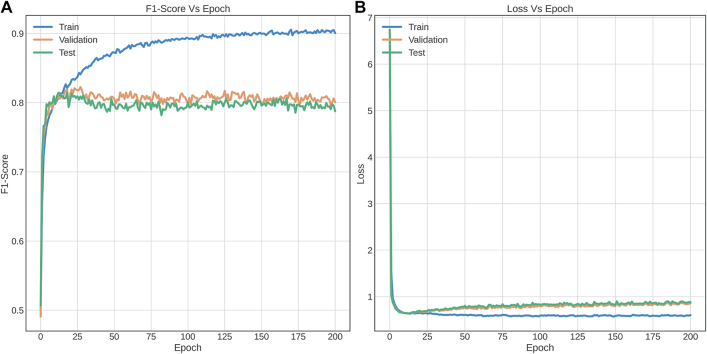
Training curves with CNN implemented for binary classification (A) F1-score vs. epochs (B) Loss vs. epochs.

**Figure 11:**
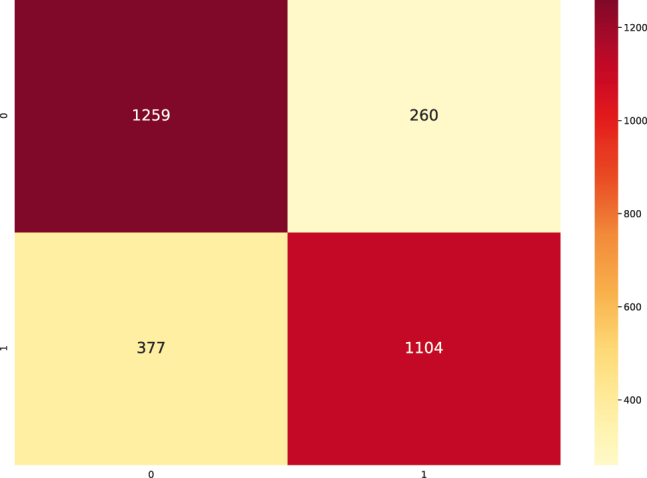
Confusion matrix made with the test dataset produced by the CNN with binary classification.

**Performance with CNN.** The corresponding experiments based on TERL model [9] were performed and the results obtained for the training curve are shown in Figure 10, with which the F1-score of 80.09% was obtained for the test data, and Figure 11 shows the confusion matrix obtained for the same data (Supplementary Material S4).

With these graphs it is identified that the model that has generalized better is the one made only with FNN, considering de two-label dataset. In addition it implies less computational expense at the time of executing it. With this model defined, we proceeded to test with genomes that have different size to verify its performance.

### 3.3 Test for the generalization of the implemented model

In order to analyse the performance of the implemented computational model, LTR retrotransposons were predicted in four different plant species: *O. indica, O. granulata, C. eugenioides* and *C. humblotiana*, using LTR_FINDER and LTR_STRUC software (Table 3).

**Table 3:** Number of predicted LTR-RTs with LTR_FINDER and LTR_STRUC software.

Genomes	Genome size	Accession number	Number of LTR-RTs	
			LTR_FINDER	LTR_STRUC
*Oryza indica*	355 Mb	GCA_011764405.2, 355	923	854
*Oryza granulata*	752 Mb	GCA_003991445.1	8597	5734
*Coffea eugenioides*	678 Mb	GCA_003713205.1	6872	3590
*Coffea humblotiana*	407 Mb	(42)	2659	2533

The different datasets created for each genome, were further processed by two workflows. The first one uses the filters implemented in InpactorDB, while the second one implement the model described above, considering the pre-processing techniques necessary for the DNA sequences to be processed correctly.

Tables 4 and 5 show the comparison of the execution times for the results of LTR_STRUC and LTR_FINDER, respectively, considering the conventional method and the computational model performed. It should be noted that the characteristics used by the model were obtained from the *k*-mers frequencies. Likewise, the number of sequences detected as non-intact elements can be seen in Table 6 for the LTR_STRUC and LTR_FINDER datasets.

**Table 4:** Execution time of the conventional method and the model implemented for LTR_STRUC data.

Genomes	Bioinformatics conventional method	DNN model	
		*K*-mer counting	FNN prediction
*Oryza indica*	00:40:12	00:00:30	00:00:3.73
*Oryza granulata*	06:04:53	00:03:13	00:00:22.61
*Coffea eugenioides*	03:03:52	00:02:01	00:00:20.59
*Coffea humblotiana*	02:07:07	00:01:27	00:00:8.5

The execution time is presented in hours:minutes:seconds.

**Table 5:** Execution time of the conventional method and the model implemented for LTR_FINDER data.

Species	Bioinformatics conventional method	DNN model	
		*K*-mer counting	FNN prediction
*Oryza indica*	00:45:14	00:00:32	00:00:11.84
*Oryza granulata*	10:09:37	00:04:49	00:00:53.01
*Coffea eugenioides*	06:51:29	00:03:50	00:00:29.01
*Coffea humblotiana*	02:05:06	00:01:30	00:00:18.09

The execution time is presented in hours:minutes:seconds.

**Table 6:** Number of sequences obtained by executing each of the methods for LTR_STRUC and LTR_FINDER data sets.

Species	LTR_SCTRUC		LTR_FINDER	
	Bioinformatics conventional	Computational model	Bioinformatics conventional	Computational model
	method		method	
*Oryza indica*	474	404	474	396
*Oryza granulata*	3266	3148	4777	4700
*Coffea eugenioides*	2436	2263	4596	4090
*Coffea humblotiana*	1630	1474	1721	1496

**Figure 12:**
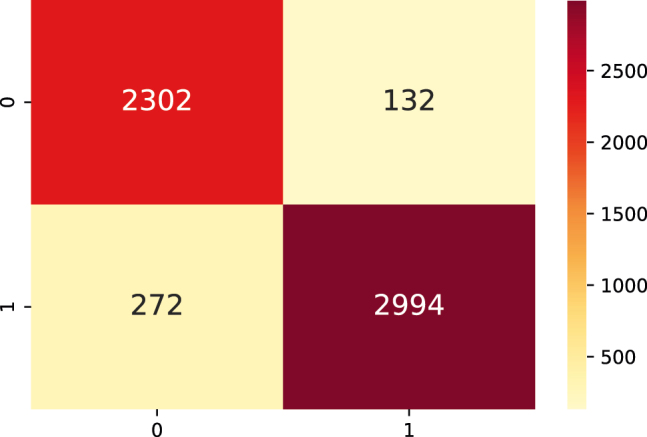
Confusion matrix performed with the generalization test dataset for the LTR_STRUC datasets of *O. granulata*.

The results obtained can be seen in Supplementary Material S5, where the best results obtained for *O. granulata* are presented in Table 7 with a F1-Score of 93.6%. Figure 12 shows the confusion matrix obtained for this dataset.

**Table 7:** Metrics obtained for the generalization test using LTR_STRUC data of *Oryza granulata*.

Metrics	Value
Run time	22.61 s
Number of filtered sequences	3148
TN (True Negative)	2302
FN (False Negative)	272
FP (False Positive)	132
TP (True Positive)	2994
Precision	0.956
Recall	0.916
Specificity/FP rate	0.704
F1-score	0.936
Accuracy	0.929

## 4 Discussion

Increasingly, machine learning is positioning itself as an effective alternative to the well-known problem of analyzing the enormous amount of sequencing data that is published every day [45]. ML has been used in a huge number of bioinformatics applications [6], [7], [8, 46, 47] demonstrating results that surpass conventional strategies, accelerating analysis times and automating tasks.

Such approaches are particularly relevant for the identification, classification and subsequent analysis of LTR retrotransposons elements in plant species [48]. In fact, this order of transposable elements represents the majority of repeated sequences in plant genomes, being able to makes up more than 50% of the genome size. The propensity of these elements to increase their copy number in genomes is directly related to their mode of transposition, that use messenger RNA in their replication mechanism [49]. Their number can be so large that they accumulate and insert themselves into each other’s, creating nested structures that are particularly difficult to identify and annotate in genome sequences [50]. Most of the tools for LTR-RT identification and reference library creation do not include automatic curation tools for these insertions. This leaves the users with a long manual curation process to identify and to remove nested insertions from LTR-RT references sequences, indicating the importance of implementing a novel tool for the automatic curation of LTR-RTs reference sequence libraries.

Currently, the most common techniques to perform this curation process is the sequence homology and structure-based approaches. Those strategies are used in conventional bioinformatics tools, in which the initial data are compared to references (proteins, domains, nucleotides) available in databases such as in REXDB [17]. However, it has disadvantages, as it requires a lot of manual work, the execution time is quite long. For this reason, despite the existence of software for the detection of nested structures [24, 25, 51] and strategies such as EDTA [29], to create libraries of good quality, we proposed a new strategy based on machine learning to identify and to filter out nested sequences that could represent “low quality” sequence in LTR-RTs reference libraries.

When running the tests with our initial dataset, it was clear that the data overlapped between the different classes, making it difficult to separate well (Figure 3). Using ML algorithms, the best F1-score percentages were obtained for MLP and KNN, for the five-label dataset with 64.8 and 63.9%, respectively, and for the two-label dataset 89.7 and 90.3%, respectively (Figure 4). It should be noted that, at the time of obtaining the values for the precision, accuracy and recall metrics of these ML models, percentages higher than 63% for the five-label dataset and higher than 89% for the two-label dataset were obtained. These results have strongly oriented the implementation towards DL architectures, since MLP is mainly based on layers of neurons.

Thus, a model was implemented that achieved an F1-score of 88.75% to identify each filter separately and 91.18% for binary detection (using the two-label dataset), highlighting in the last one, the values obtained for precision, accuracy and recall, which are 91.40, 91.25 and 91.25% respectively (Table 2), percentages that were obtained from the implementation of a computational tool that runs in seconds. It is emphasized that those non-intact sequences should be disregarded from the final dataset and stored in new files, because for future studies these sequences are of great relevance, to observe the divergence and establish an evolutionary scale of the analyzed species.

Finally, generalization tests were performed with four plants genomes: *C. eugenioides* (678 Mb), *C. humblotiana* (407 Mb), *O. indica* (355 Mb) and *O. granulata* (752 Mb), which have a number of predicted LTR-RTs of 3,590, 2,533, 854 and 5734 respectively (Table 3), according to the results of LTR_STRUC. A percentage higher than 85% was obtained in all cases. Interestingly, the execution time is greatly reduced from hours to seconds with the implemented FNN when compared to conventional methods (Tables 4 and 5).

However, the results obtained for the LTR_FINDER dataset range between 54.8 and 59.2% for the F1-score percentage. This significantly lower percentages when compared to LTR_STRUC, can be attributed to a highest rate of false positives.

Altogether our results indicate that the implementation of FNN method for curation of LTR-RTs sequence is relevant, optimizing the execution time for the creation of better quality reference libraries for plant genomes. This model can be integrated into existing tools as an extra filter due to the short running time and low computational resources needed to make the predictions. Due to the sequential behaviour of the data used in this work, better results could be obtained by using recurrent neural networks, since these have a memory that allows storing key information. As a future work, we will use the methodology proposed in this work (dataset, pre-processing and feature extraction) but using recurrent networks such as LSTM (Long-Short term Memory) or transformers in order to compare the performance against the FNN and CNN networks implemented in this work.

## 5 Conclusions

With the sequencing and release of large amounts of genomes currently underway, the creation of more efficient run-time methodology is a necessity. Neural networks can be used to solve bottlenecks in genomic processes and the results proposed in this work demonstrate this once again. The FNN-based model obtained 91.18% F1-Score for detecting LTR retrotransposon sequences with nested structures. In addition, curation of an *O. granulata* library was achieved with an F1-score of 93.6% and an approximate network prediction time of 22.61 s.

## Supplementary Material

Supplementary Material Details

Supplementary Material Details

Supplementary Material Details

Supplementary Material Details

Supplementary Material Details
