# Supplementary material for: Automatic curation of LTR retrotransposon libraries from plant genomes through machine learning
Source: J Integr Bioinform. 2022 Jul 12;19(3):20210036. doi: 10.1515/jib-2021-0036 (PMC9521825; doi:10.1515/jib-2021-0036)

Dataset	Metrics	Scaling+ PCA							
		LR	LDA	KNN	MLP	RF	DT	NB	SVC
		C=0,899	tol=0,0001	n_neighbors=1	hidden_layer_size= 250	n_estimators= 70	max_depth=9	var_smoothing= 0,1	C=90
5 labels	F1-score	0,559967	0,564487	0,639990	0,648718	0,507465	0,457652	0,197348	0,513147
	Accuracy	0,835889	0,822497	0,863761	0,869629	0,824910	0,779529	0,259559	0,839201
	Recall	0,519248	0,531627	0,635461	0,630141	0,461068	0,429513	0,309203	0,475454
	Precision	0,670247	0,651569	0,645724	0,655328	0,706091	0,604595	0,429123	0,743394
		LR	LDA	KNN	MLP	RF	DT	NB	SVC
		C=0,999	tol=0,0001	n_neighbors=11	hidden_layer_size= 350	n_estimators= 80	max_depth=9	var_smoothing= 1e-07	C=90
2 labels	F1-score	0,841991	0,841900	0,897689	0,903918	0,870237	0,827852	0,571977	0,861668
	Accuracy	0,843318	0,843555	0,898968	0,904505	0,869724	0,830825	0,574248	0,863903
	Recall	0,841039	0,840521	0,895538	0,903274	0,863964	0,825535	0,582292	0,859212
	Precision	0,843706	0,845020	0,903004	0,904675	0,879625	0,836828	0,585899	0,869891

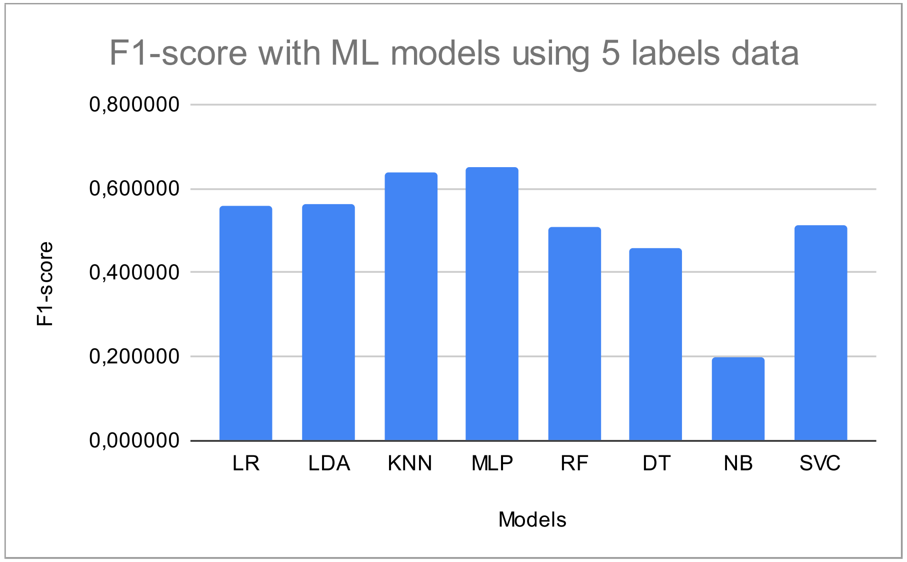

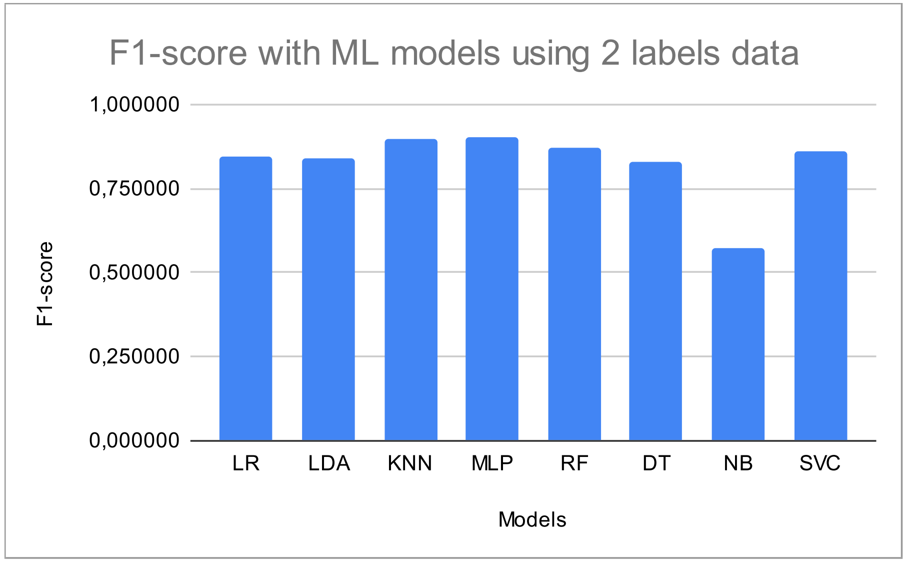

Supplement: Supplementary file 1 — Supplementary Material Details [file j_jib-2021-0036_suppl_001.pdf]
