# Supplementary material for: Automatic curation of LTR retrotransposon libraries from plant genomes through machine learning
Source: J Integr Bioinform. 2022 Jul 12;19(3):20210036. doi: 10.1515/jib-2021-0036 (PMC9521825; doi:10.1515/jib-2021-0036)
Supplement: Supplementary file 2 — Supplementary Material Details [file j_jib-2021-0036_suppl_002.pdf]

Primeras Pruebas - Deep Learning

Capas	Neuronas	F1-score	Loss	Gráfica F1-score vs épocas	Gráfica F1-score vs épocas	Gráfica pérdida vs épocas	Matriz de confusión Train	Matriz de confusión Validation	Matriz de confusión Test
1	200	0,8762	0,7849	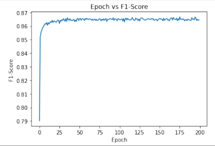	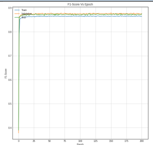	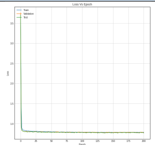	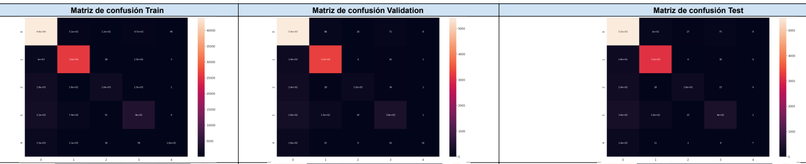		
2	200	0,876	0,7792	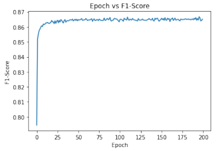	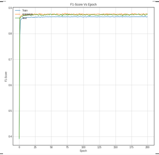	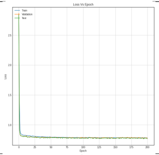	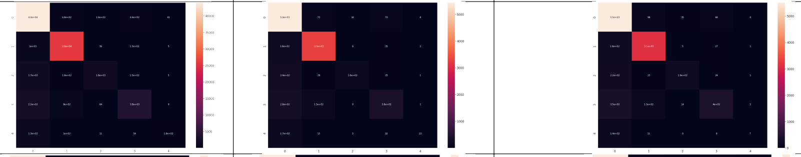		
3	100	0,8808	0,7556	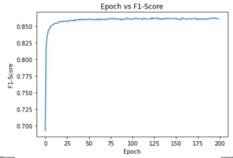	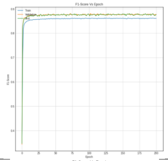	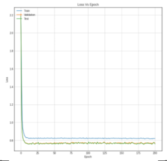	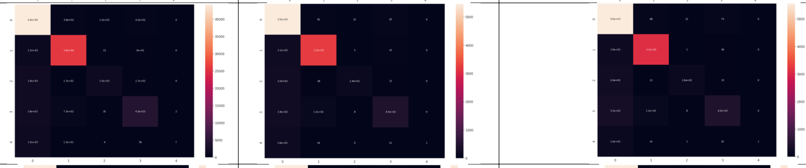		
	200	0,8875	0,9139	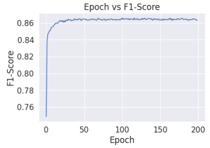	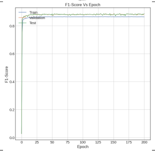	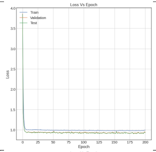	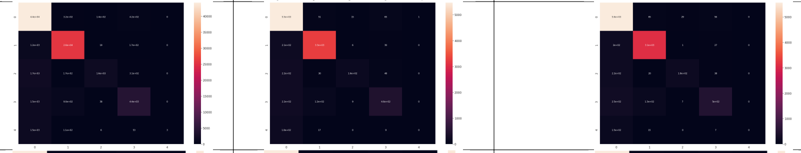		
	300	0,8872	1,0472	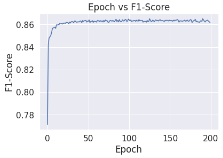	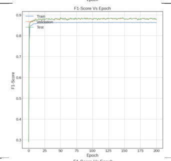	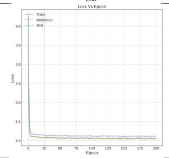	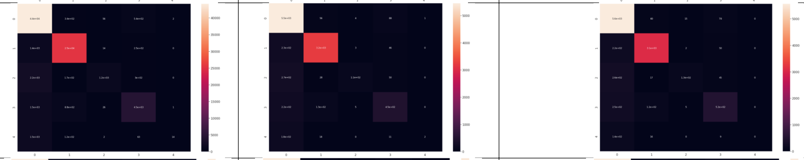		
	400	0,8852	1.1768	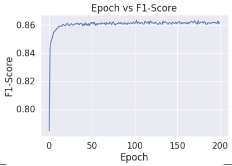	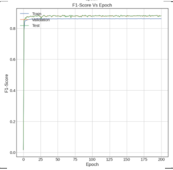	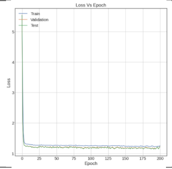	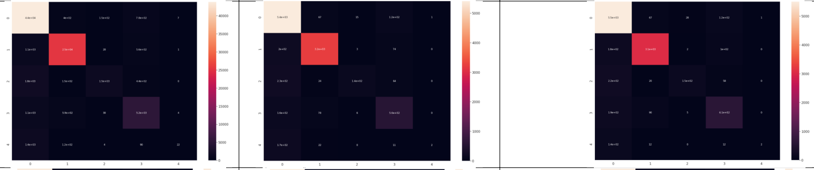		
	500	0,8840	1.2857	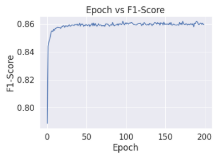	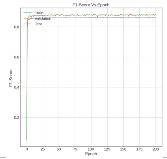	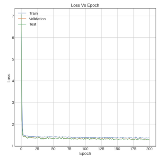	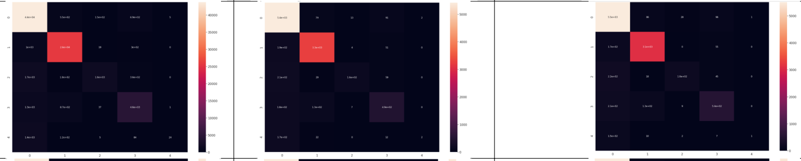		
	600	0,8829	1,4091	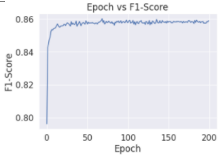	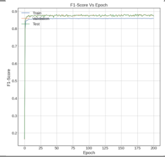	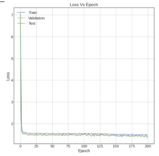	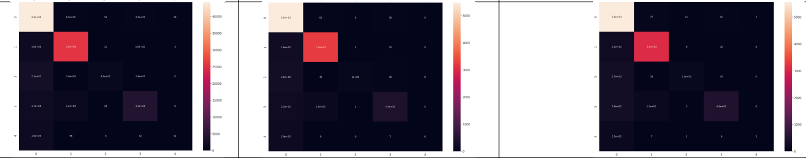		

	700	0,8806	1,5481	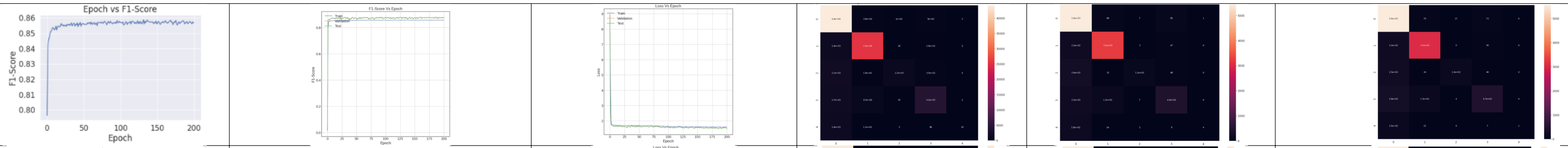
	800	0,881	1,7266	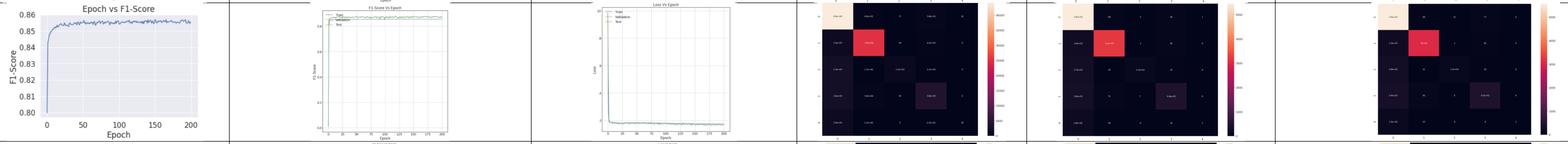
4	200	0,8838	0,9675	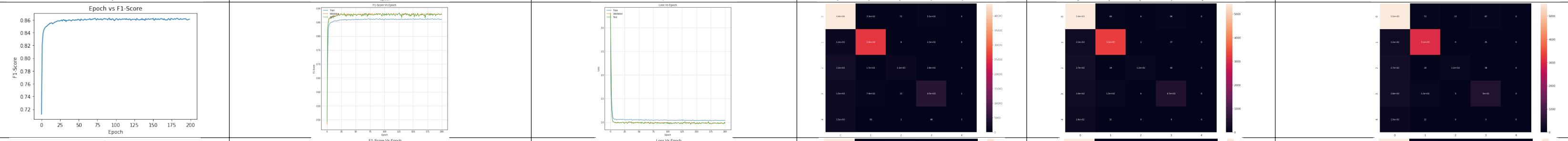
5	200	0,8812	1,0209	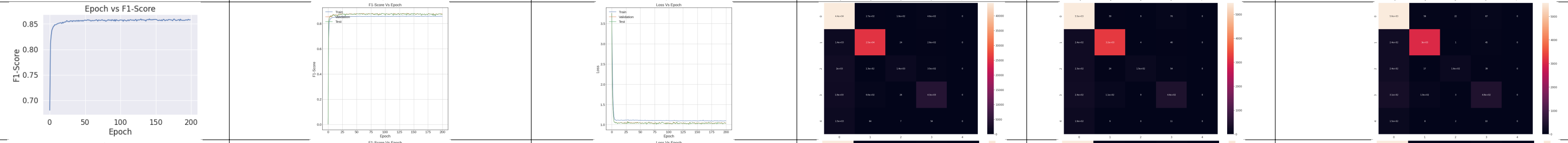
6	200	0,8818	1,0603	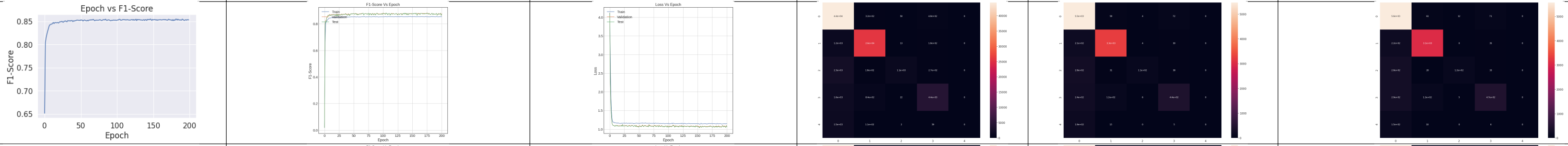
7	200	0,8781	1,1427	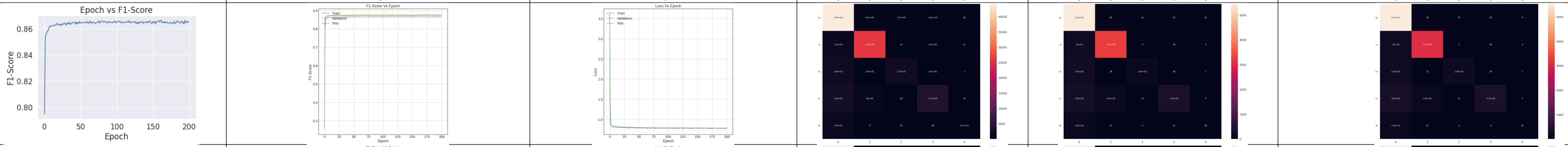
8	200	0,8758	1,1492	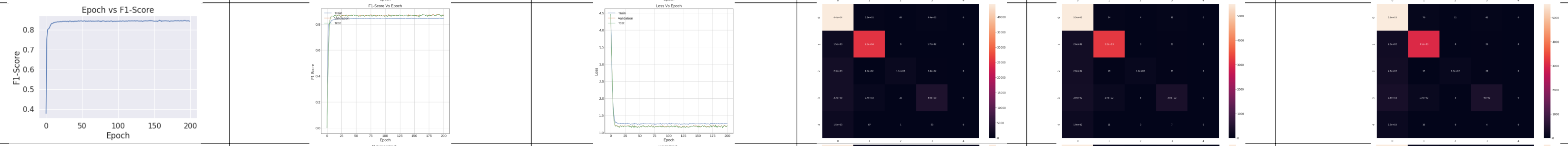
9	200	0,871	1,2277	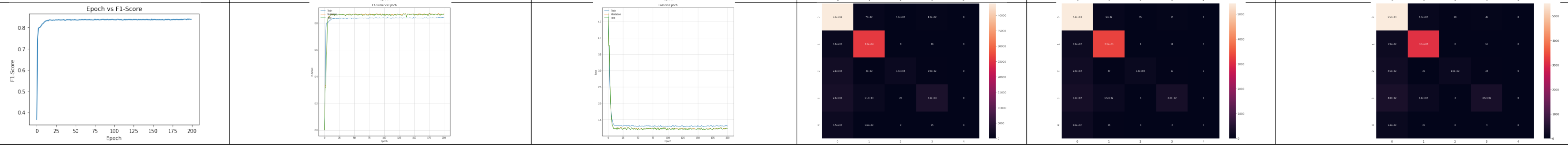

10	200	0,8687	1,2618	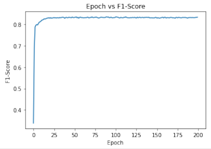	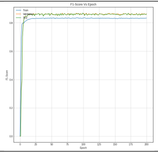	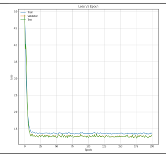	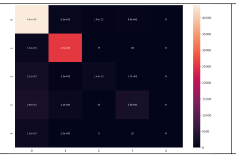	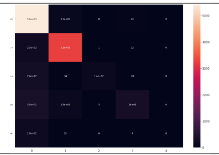	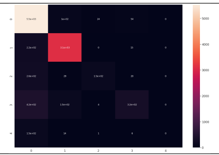
