# Supplementary material for: Automatic curation of LTR retrotransposon libraries from plant genomes through machine learning
Source: J Integr Bioinform. 2022 Jul 12;19(3):20210036. doi: 10.1515/jib-2021-0036 (PMC9521825; doi:10.1515/jib-2021-0036)
Supplement: Supplementary file 3 — Supplementary Material Details [file j_jib-2021-0036_suppl_003.pdf]

Capas	Neuronas	F1-score	Loss	Gráfica F1-score vs épocas	Gráfica F1-score vs épocas	Gráfica pérdida vs épocas	Matriz de confusión Train	Matriz de confusión Validation	Matriz de confusión Test
8	1400-100	0,8985	2,0774	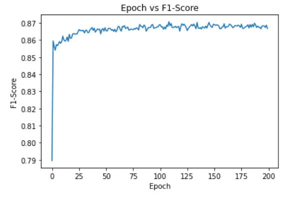	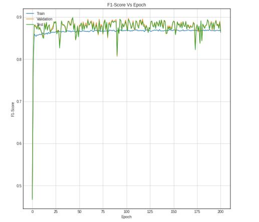	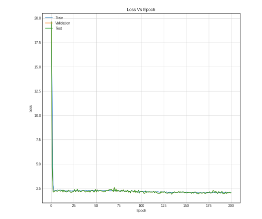	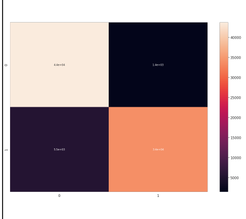	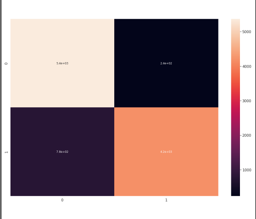	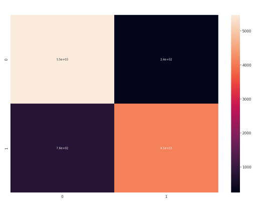
4	300	0,9112	0,7539	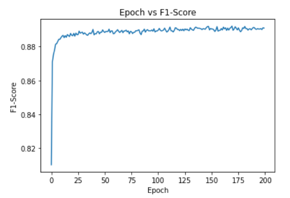	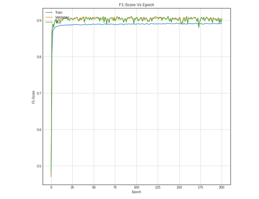	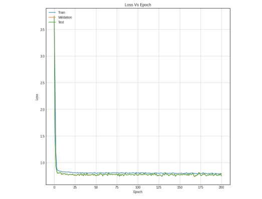	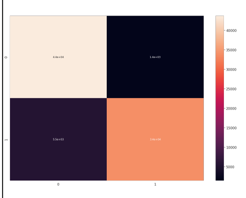	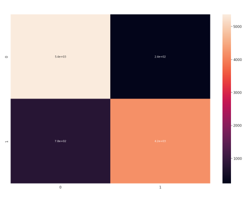	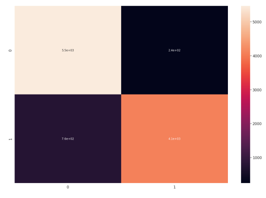
6	400-1400	0,9079	1,6891	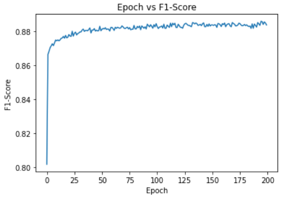	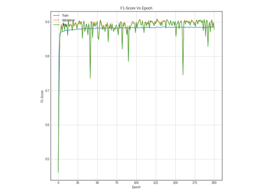	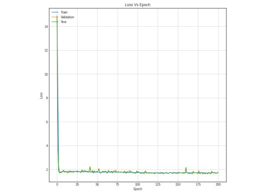	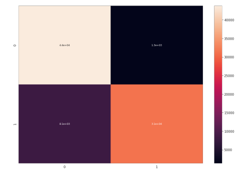	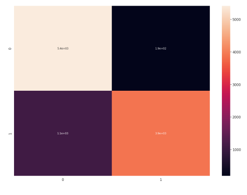	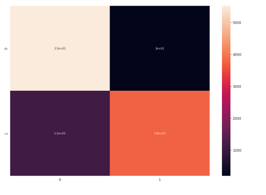
3	400	0,909	0,8192	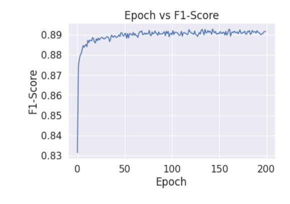	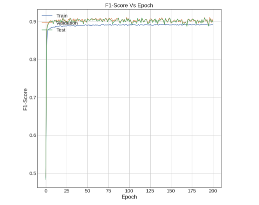	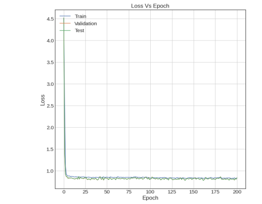	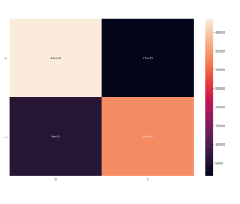	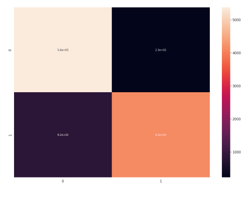	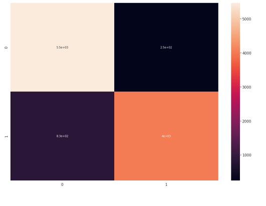
3	200	0,9118	0,607	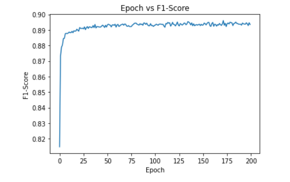	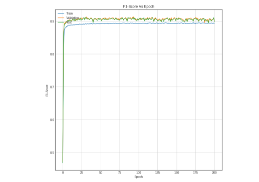	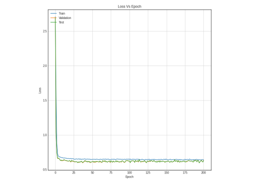	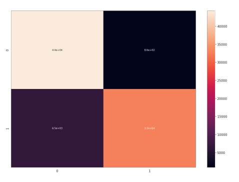	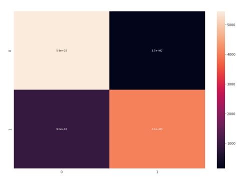	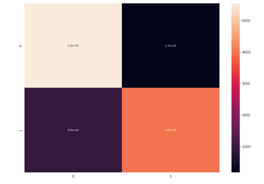
8	200	0,9107	0,7587	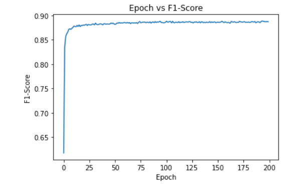	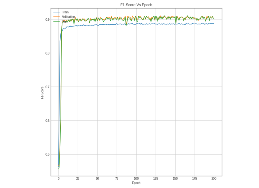	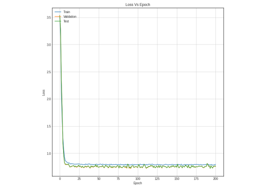	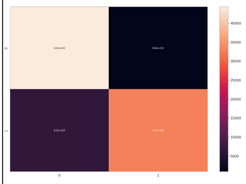	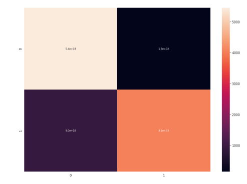	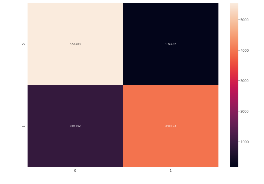

PRUEBA COMPORTAMIENTO "AUTOENCODER"										
Relación	Capas	Neuronas	F1-score	Loss	Gráfica F1-score vs épocas	Gráfica F1-score vs épocas	Gráfica pérdida vs épocas	Matriz de confusión Train	Matriz de confusión Validation	Matriz de confusión Test
Nota adjunta [1]	22	800 a 800	0,8837	2,4616	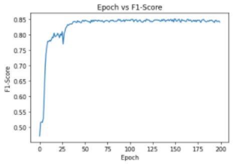	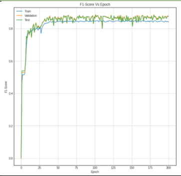	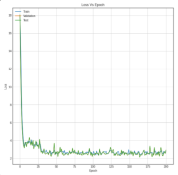	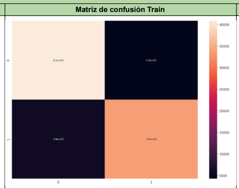	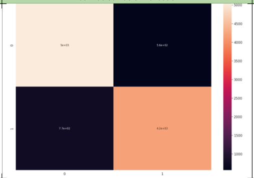	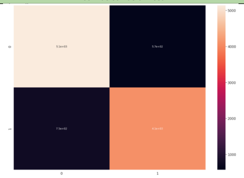
[2]	20	700 a 700	0,8995	1,7144	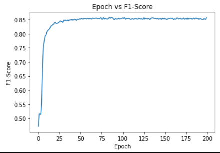	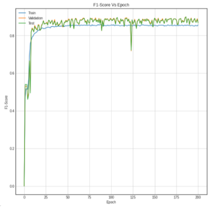	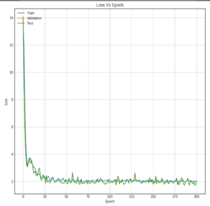	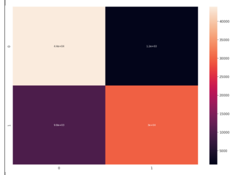	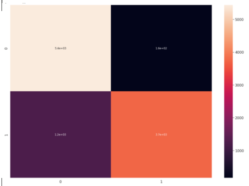	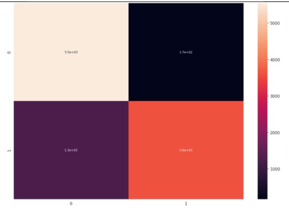
[3]	18	600 a 600	0,8993	1,5414	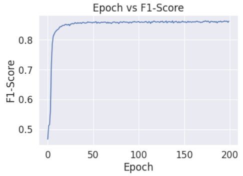	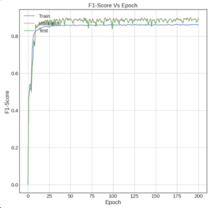	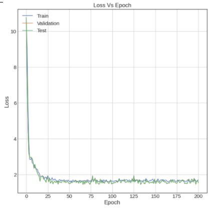	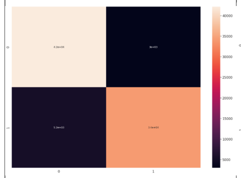	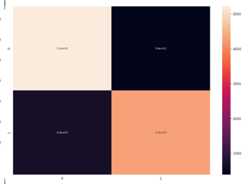	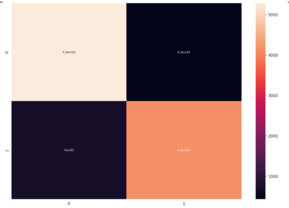
[4]	16	500 a 500	0,9027	1,3358	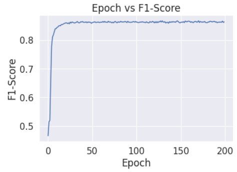	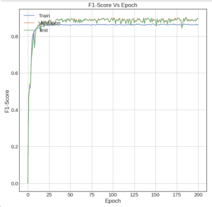	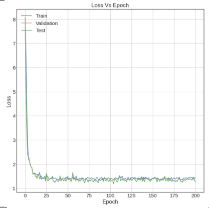	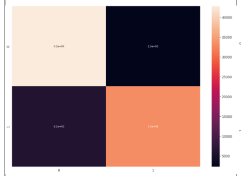	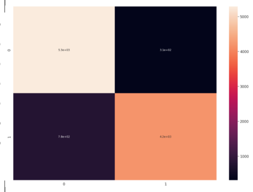	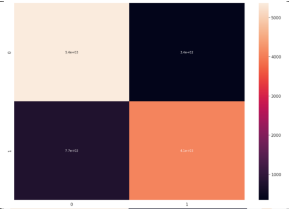
[5]	14	400 a 400	0,9086	0,9307	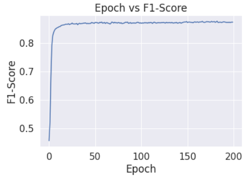	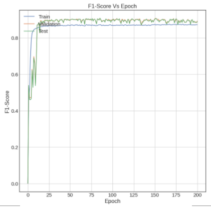	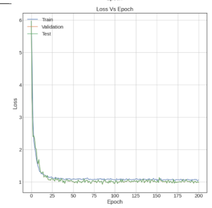	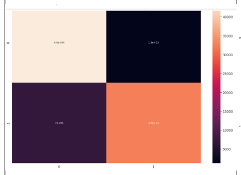	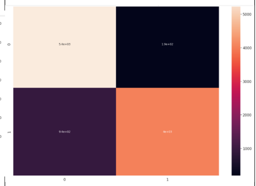	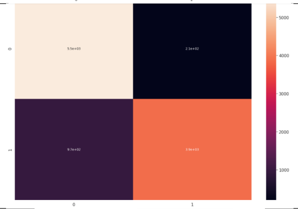
[6]	12	300 a 300	0,911	0,7861	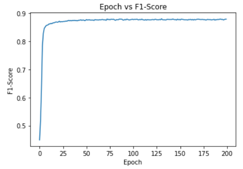	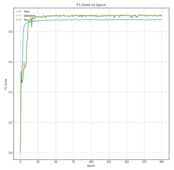	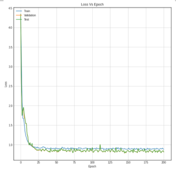	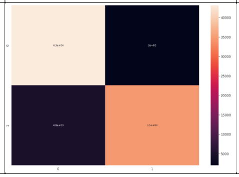	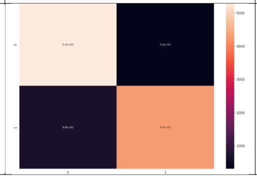	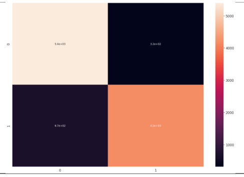

[7]	10	200 a 200	0,9077	0,6874	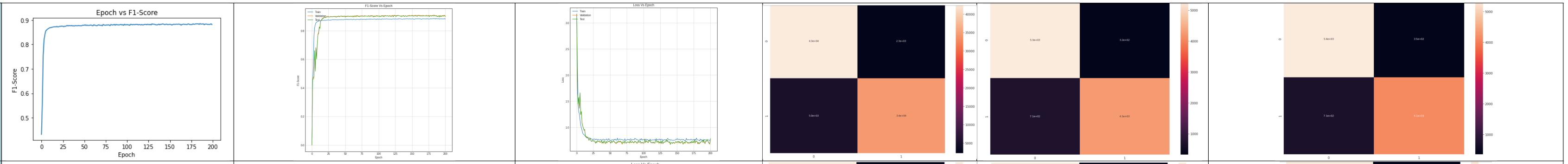					
[8]	8	100 a 100	0,9036	0,5767	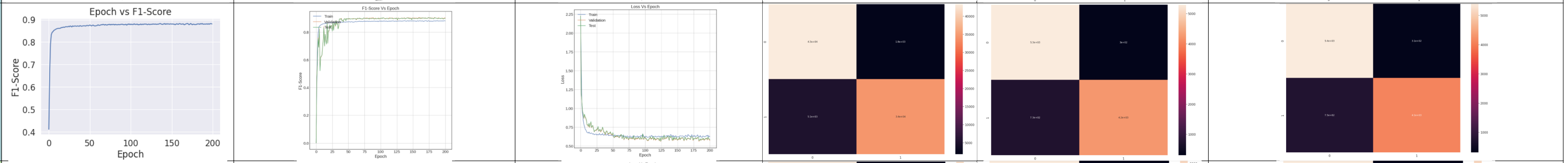					
Nota adjunta [9]	22	400 - 400	0,8999	0,5313	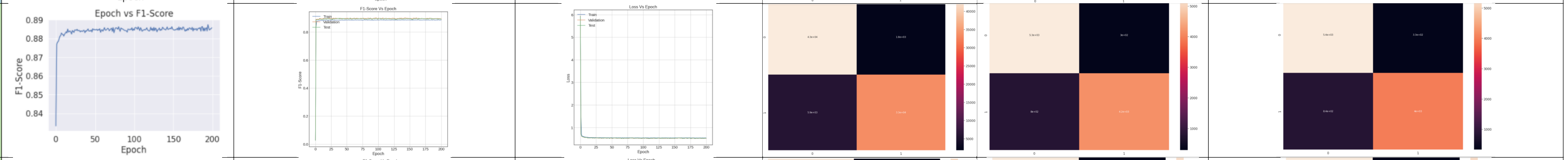					
[10]	20	350 - 350	0,9012	0,503	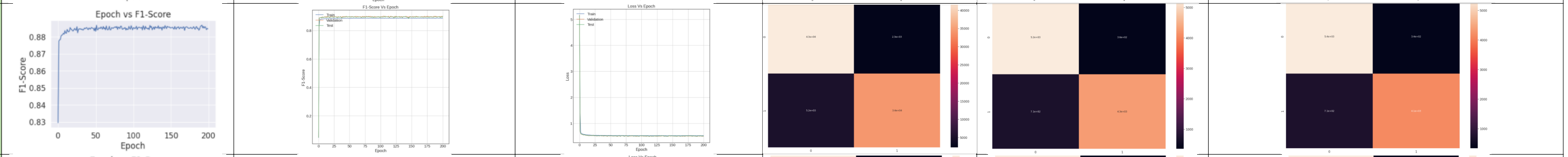					
	18	300 - 300	0,901	0,4973	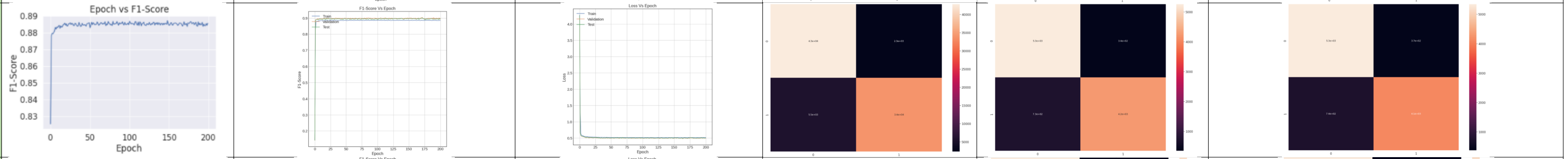					
	16	250 - 250	0,8989	0,4781	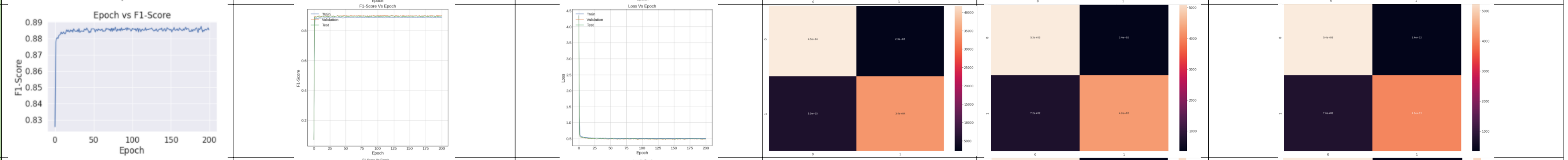					
	14	200 - 200	0,8988	0,4768	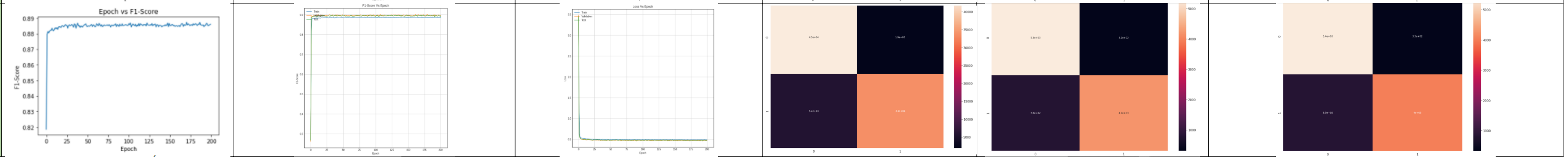					

	12	150 - 150	0,8979	0,4606	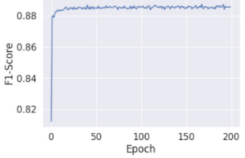	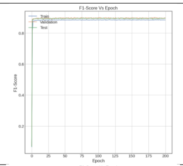	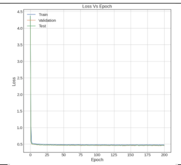	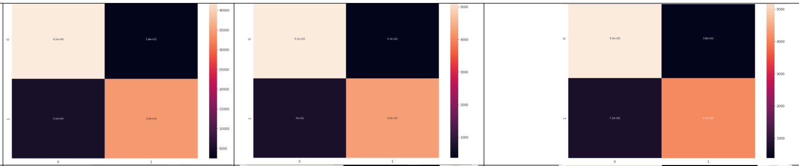		
	10	100 - 100	0,9103	0,4679	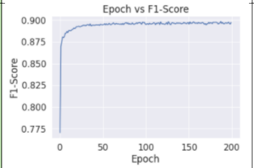	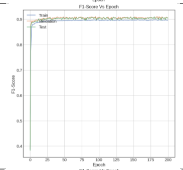	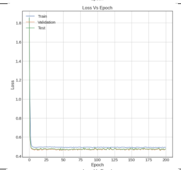	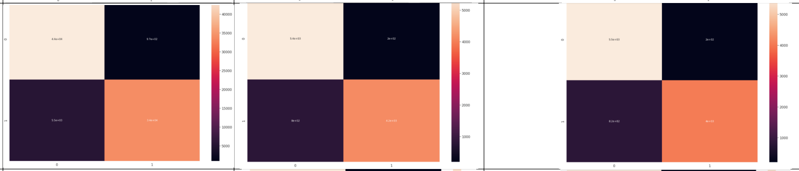		
	8	75 - 75	0,8988	0,4272	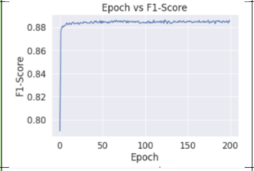	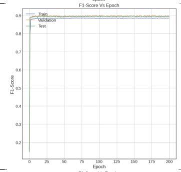	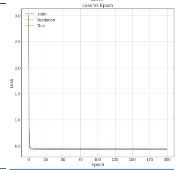	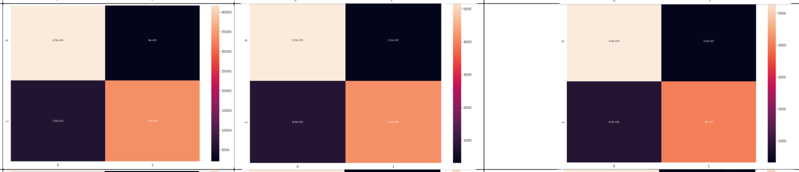		
	6	50 - 50	0,8671	0,5231	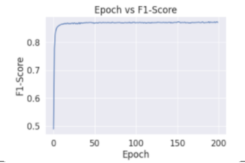	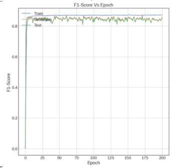	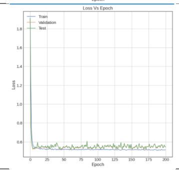	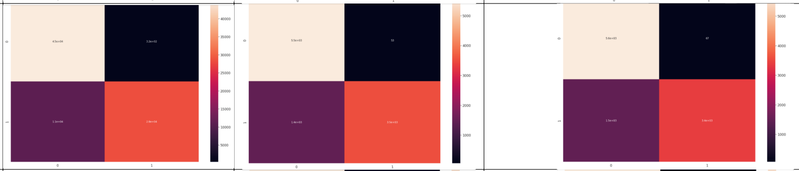		
Nota adjunta [11]	26	1600-1600	0,8973	0,7577	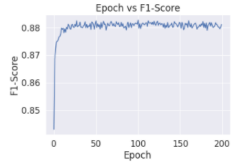	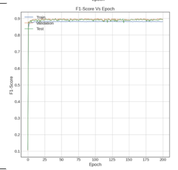	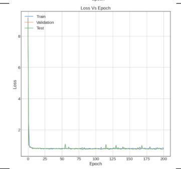	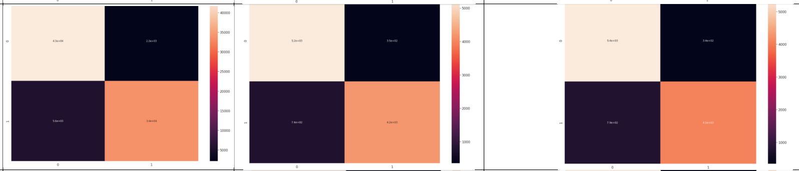		
	24	1400-1400	0,8976	0,7318	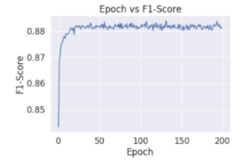	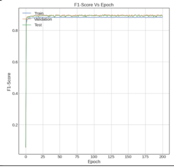	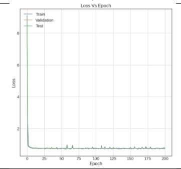	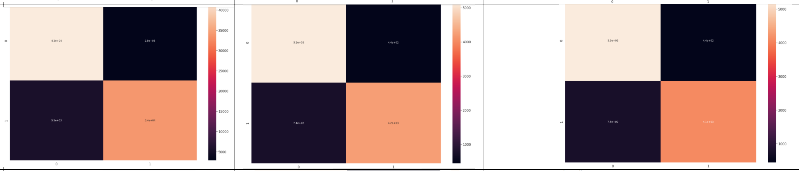		
	22	1200 - 1200	0,8984	0,6615	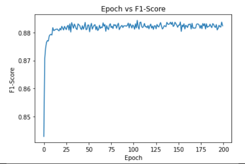	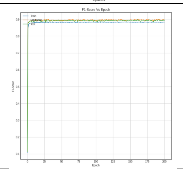	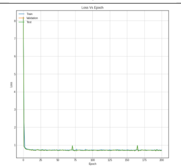	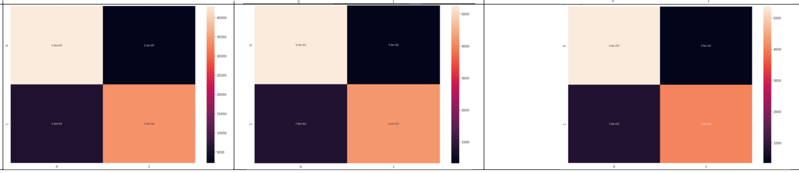		

		20	1000 - 1000	0,8987	0,6569	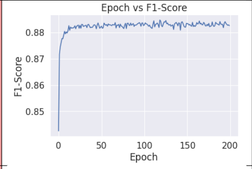	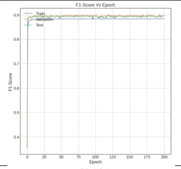	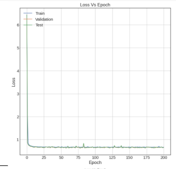	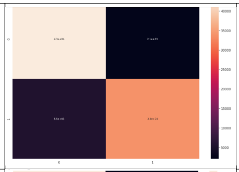	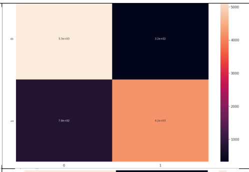	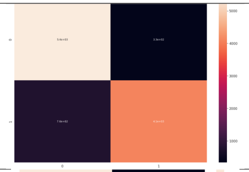
		18	800 - 800	0,898	0,6056	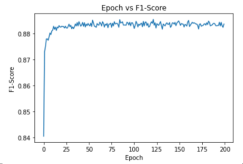	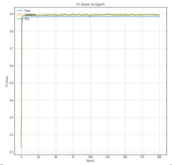	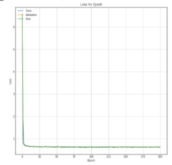	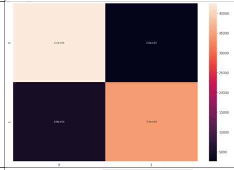	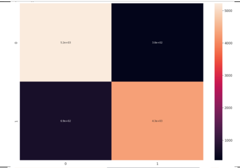	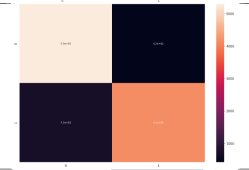
		16	600 - 600	0,8972	0,5589	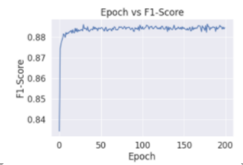	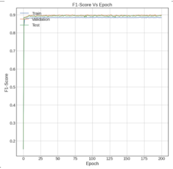	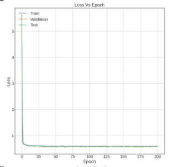	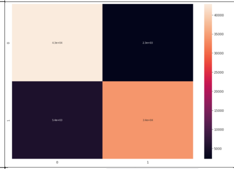	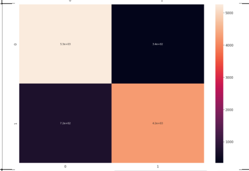	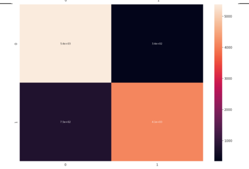
		14	400 - 400	0,9006	0,5281	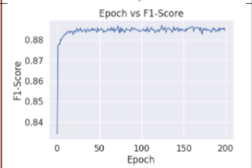	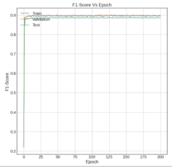	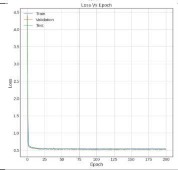	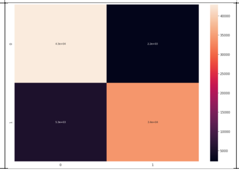	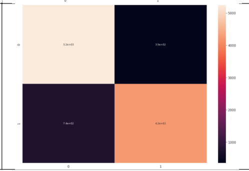	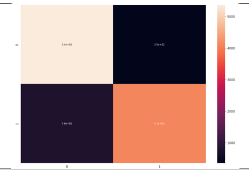

Función de pérdida: "Binary Crossentropy"									
Capas	Neuronas	F1-score	Loss	Gráfica F1-score vs épocas	Gráfica F1-score vs épocas	Gráfica pérdida vs épocas	Matriz de confusión Train	Matriz de confusión Validation	Matriz de confusión Test
5	1200-200	0,8877	1,4643	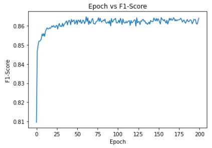	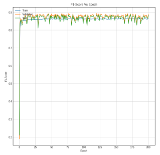	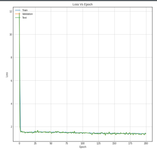	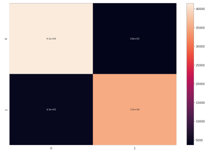	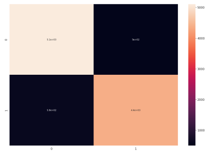	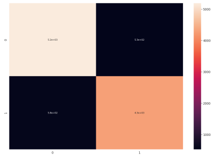
7	1200-100	0,8851	1,7267	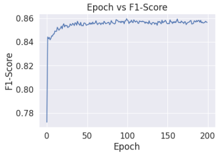	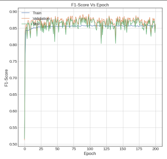	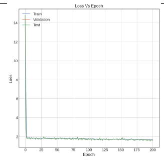	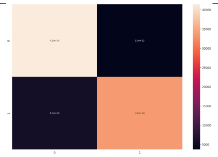	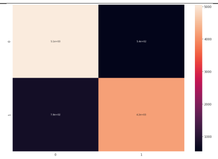	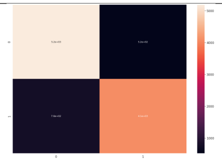
8	1400-100	0,8792	2,0756	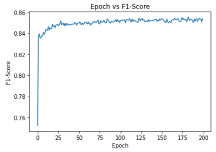	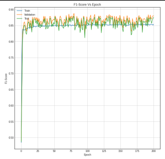	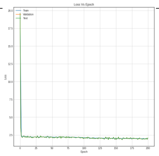	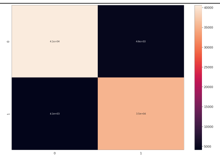	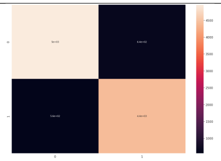	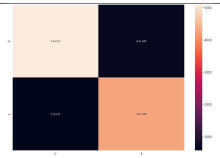
9	1600-100	0,879	2,4968	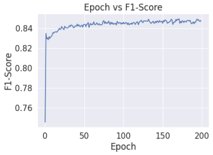	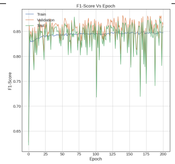	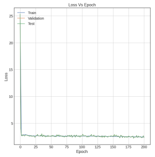	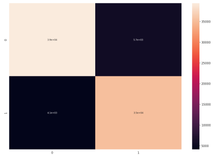	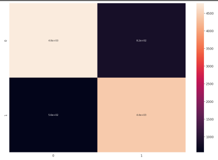	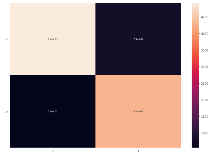
8	1600-200	0,8771	2,412	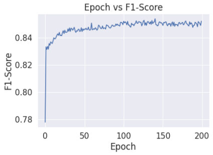	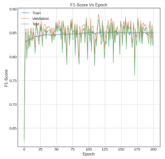	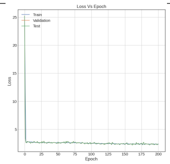	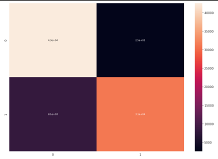	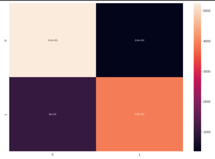	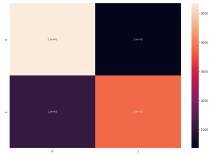
11	1600-25	0,8688	2,6229	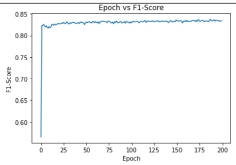	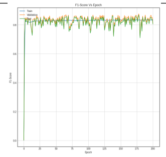	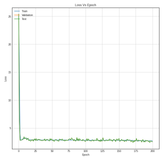	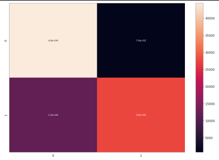	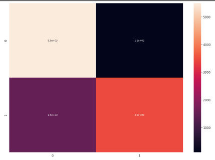	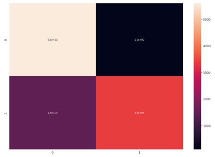

[1] Se comienza con 800 disminuyendo de 100 en 100 hasta llegar a 100, luego 50, 25, 10, 25, 50 hasta llegar de nuevo a 100 y repetir el primer intervalo hasta 800 de nuevo

[2] Se comienza con 700 disminuyendo de 100 en 100 hasta llegar a 100, luego 50, 25, 10, 25, 50 hasta llegar de nuevo a 100, y repetir el primer intervalo hasta 700 de nuevo

[3] Se sigue la misma técnica de la celda anterior

[4] Se sigue la misma técnica de la celda anterior

[5] Se sigue la misma técnica de la celda anterior

[6] Se sigue la misma técnica de la celda anterior

[7] Se sigue la misma técnica de la celda anterior

[8] Se sigue la misma técnica de la celda anterior

[9] Se comienza con 400 disminuyendo de 50 en 50 hasta llegar a 100, luego 75, 50, 25, 10, 25, 50, 75 hasta llegar de nuevo a 100 y repetir el primer intervalo hasta 400 de nuevo

[10] Se comienza con 350 disminuyendo de 50 en 50 hasta llegar a 100, luego 75, 50, 25, 10, 25, 50, 75 hasta llegar de nuevo a 100 y repetir el primer intervalo hasta 350 de nuevo

[11] Se comienza con 1600 disminuyendo de 200 en 200 hasta llegar a 200, luego 100, 75, 50, 25, 10, 25, 50, 75, 100 hasta llegar de nuevo a 200 y repetir el primer intervalo hasta 1600 de nuevo
