# Supplementary material for: Automatic curation of LTR retrotransposon libraries from plant genomes through machine learning
Source: J Integr Bioinform. 2022 Jul 12;19(3):20210036. doi: 10.1515/jib-2021-0036 (PMC9521825; doi:10.1515/jib-2021-0036)
Supplement: Supplementary file 4 — Supplementary Material Details [file j_jib-2021-0036_suppl_004.pdf]

CNN con una muestra de 10.00 secuencias										
**Convoluciones**	**Capas**	**Neuronas**	**F1-score**	**Loss**	**Gráfica F1-score vs épocas**	**Gráfica F1-score vs épocas**	**Gráfica pérdida vs épocas**	**Matriz de confusión Train**	**Matriz de confusión Validation**	**Matriz de confusión Test**
3	2	1000-500	0,877	0,8942	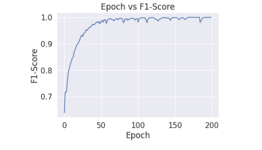	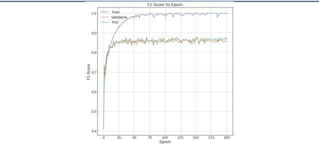	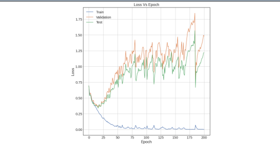	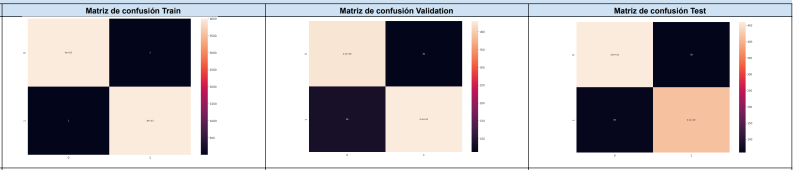		
	5	500	0,877	0,6621	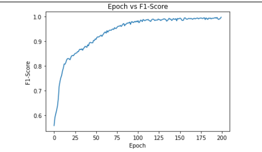	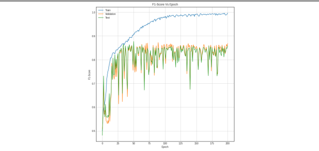	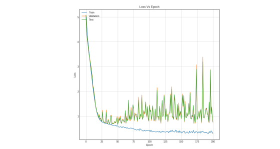	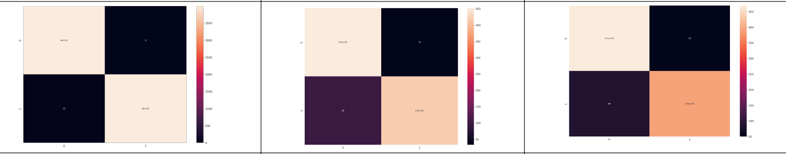		
	7	500	0,8809	1,1933	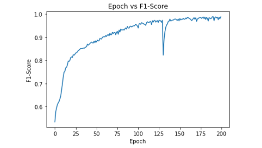	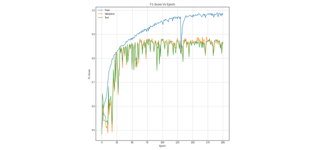	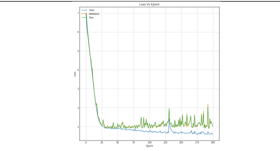	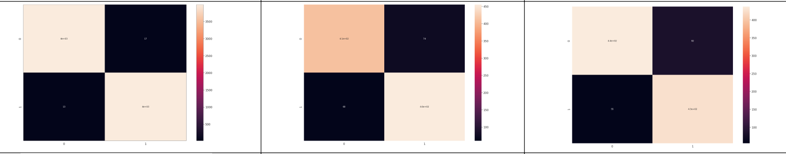		
	9	500	0,8682	1,6948	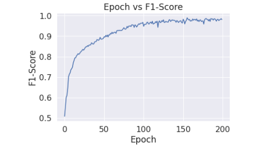	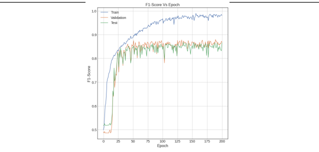	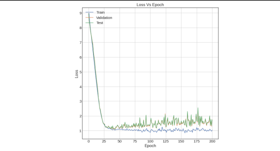	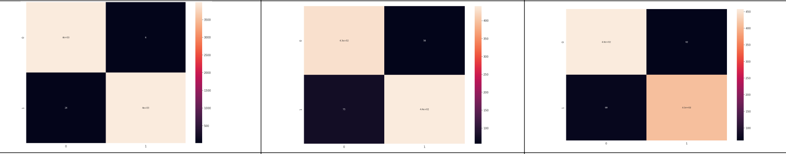		
	11	500			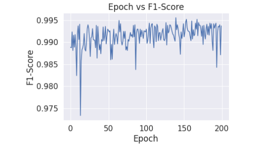	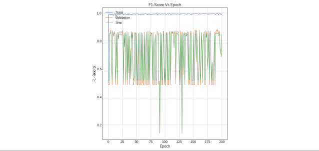	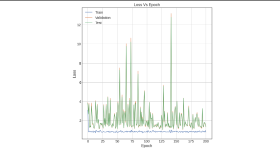	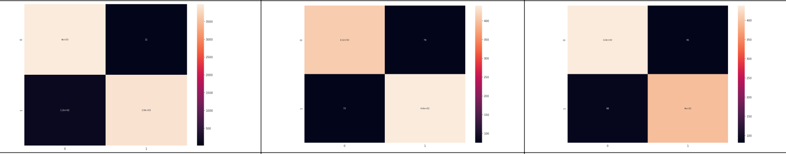		
	5	1000-600	0,8770	0,945	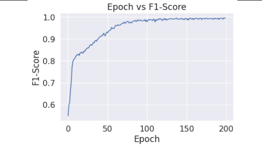	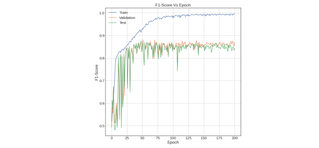	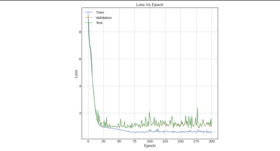	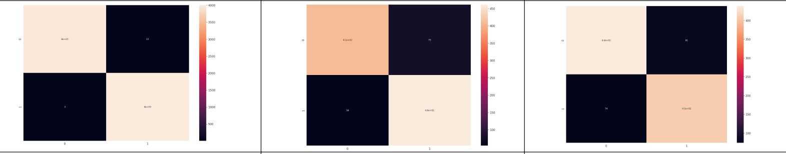		
	5	1000-200	0,8740	1,1581	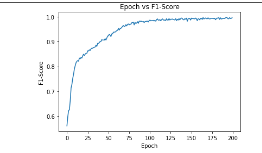	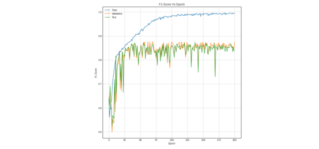	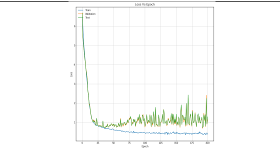	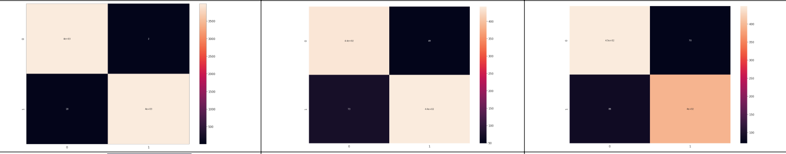		
	9	1000-200	0,876	0,991	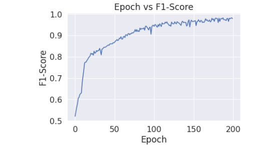	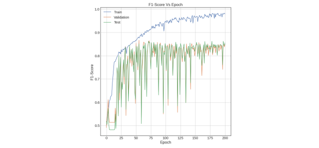	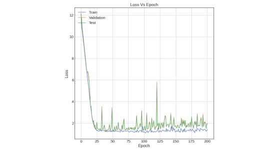	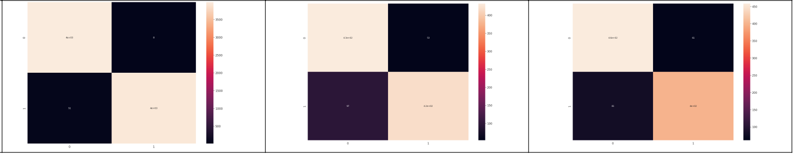		

5	2	1000-500	0,8779	0,9552	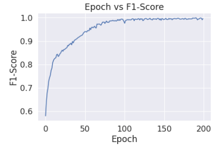	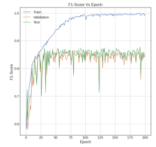	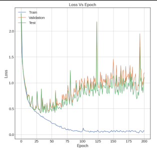	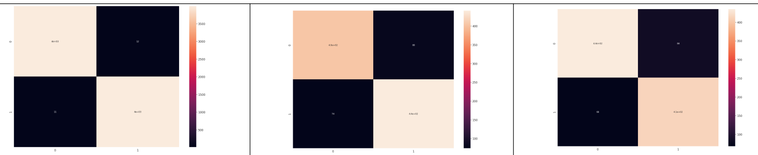		
	5	500	0,877	0,6048	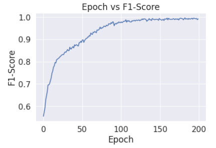	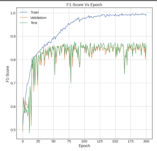	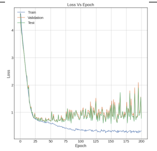	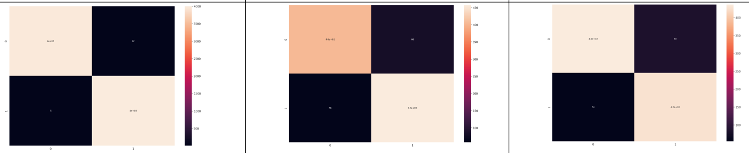		
	9	500	0,876	1,2597	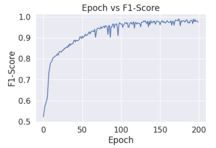	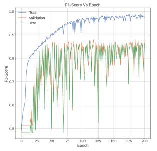	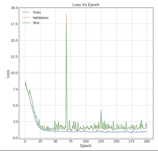	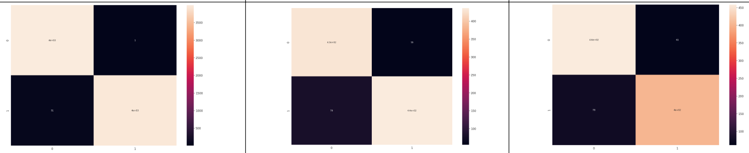		
	11	500	0,873	1,354	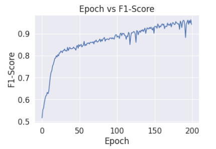	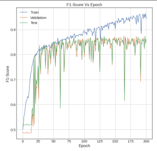	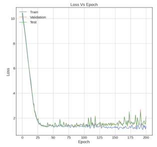	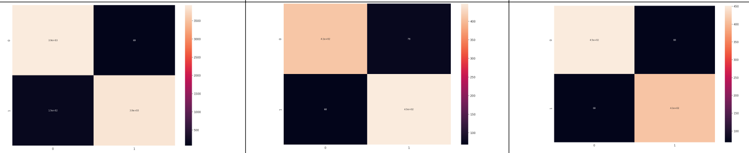		
	5	1000-600	0,8740	0,8683	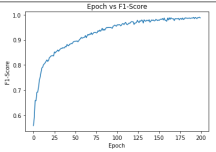	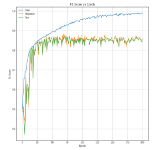	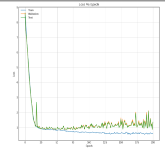	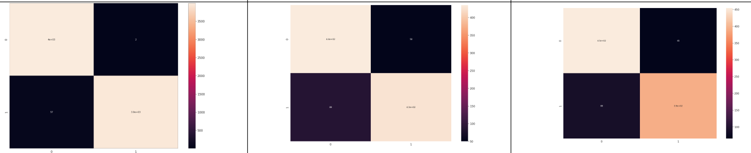		
	5	1000-200			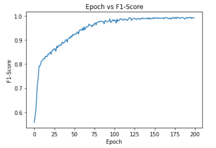	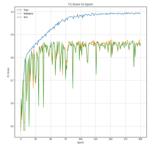	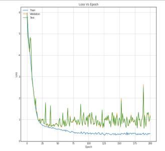	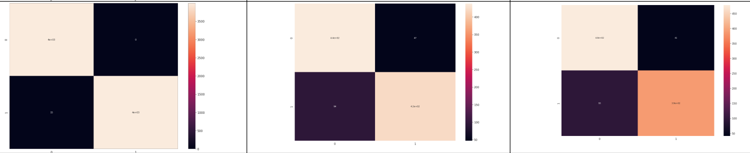		
	9	1000-200	0,8818	1,5359	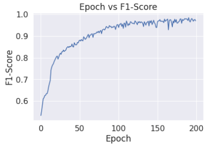	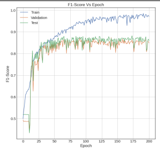	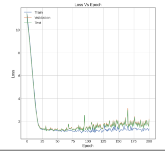	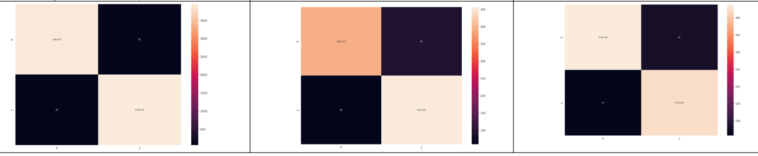		

Convoluciones	Capas	Neuronas	F1-score	Loss	Gráfica F1-score vs épocas	Gráfica F1-score vs épocas	Gráfica pérdida vs épocas	Matriz de confusión Train	Matriz de confusión Validation	Matriz de confusión Test
3	3	200 [1]	0,8623	0,3366	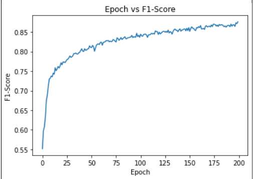	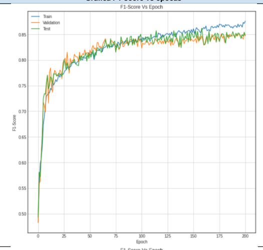	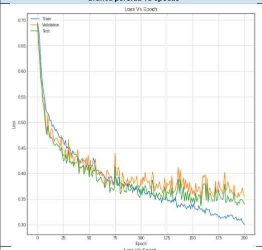	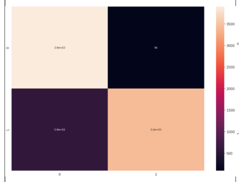	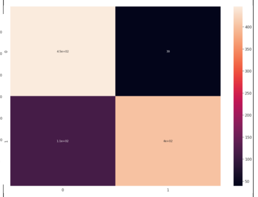	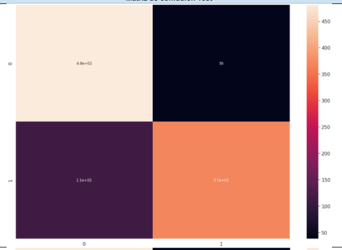
3	3	200 [2]	0,8672	0,3651	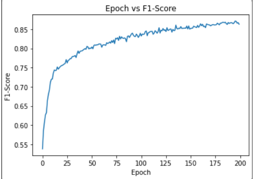	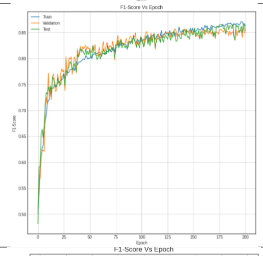	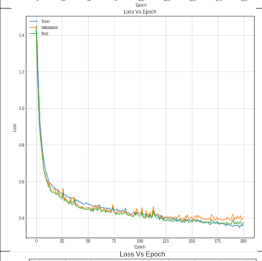	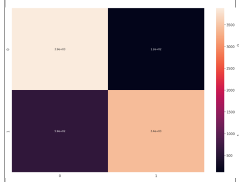	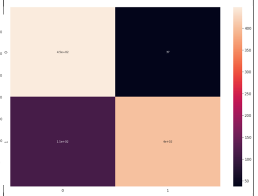	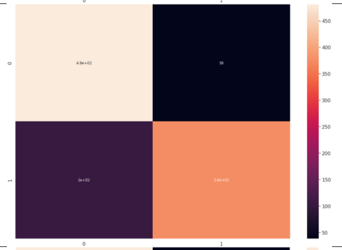
3 [3]	3	200 [4]	0,8467	0,4318	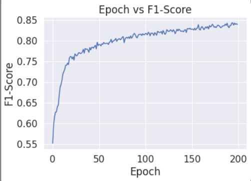	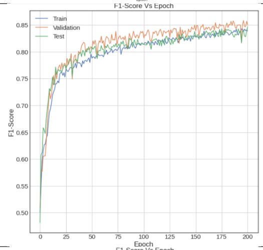	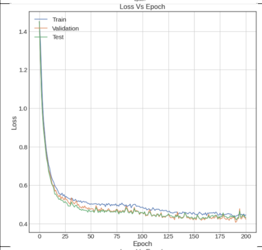	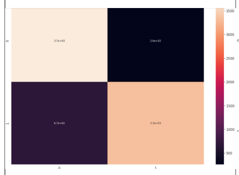	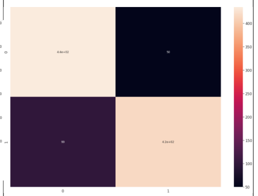	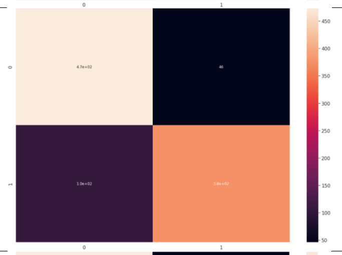
3 [5]	3	200 [6]	0,8770	0,3924	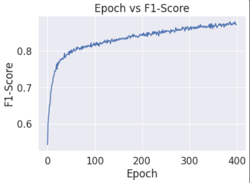	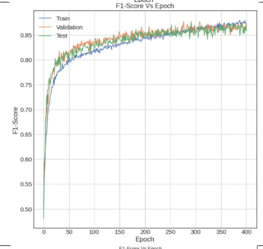	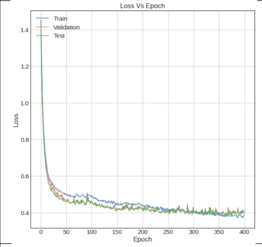	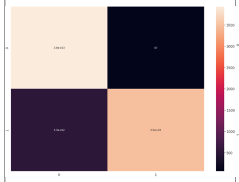	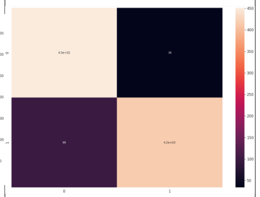	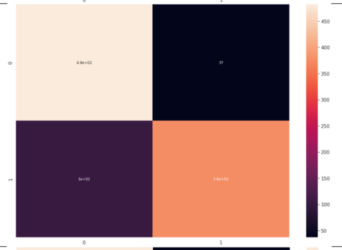
3	3	200	0,8848	0,3825	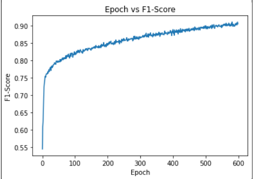	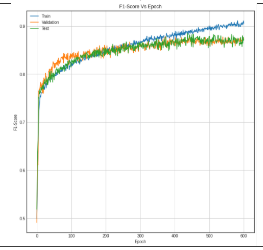	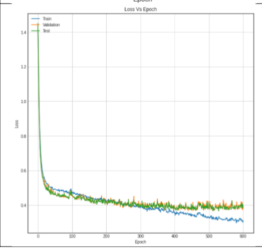	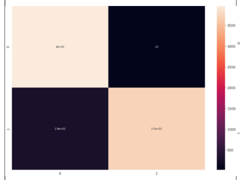	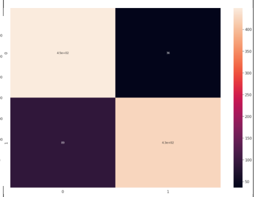	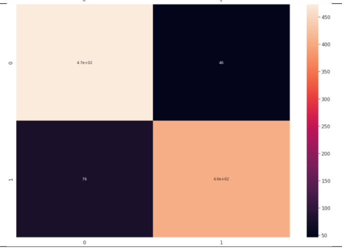

3 [7]	3	200 [8]	0,8496	0,4302	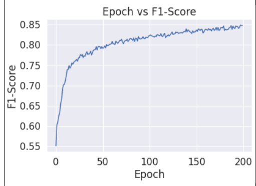	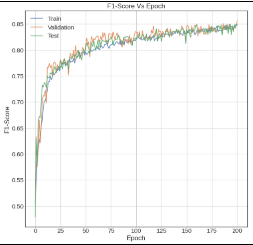	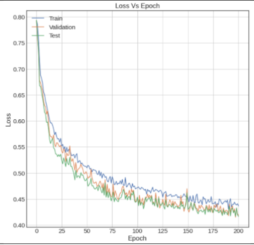	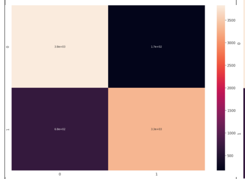	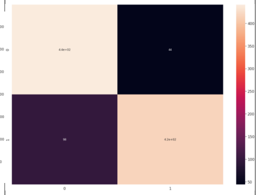	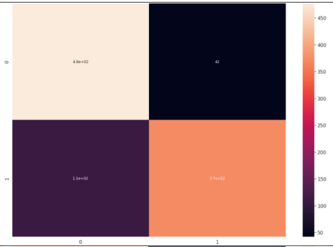
3 [9]	11	250-250 [10]	0,8408	0,4896	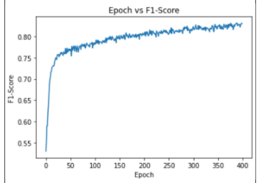	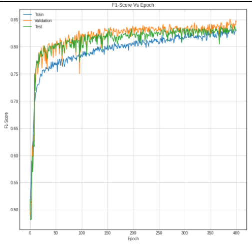	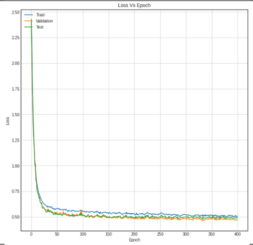	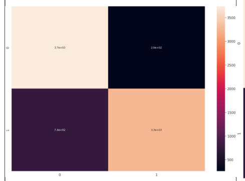	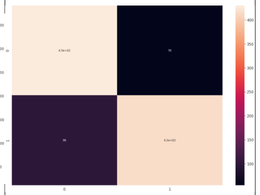	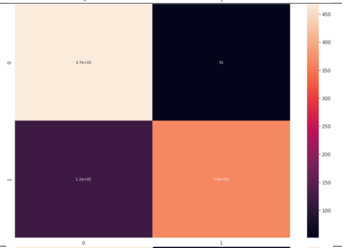
3 [11]	3	200 [12]	0,8135	0,5311	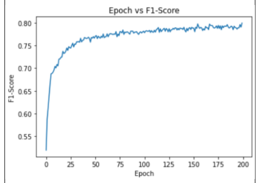	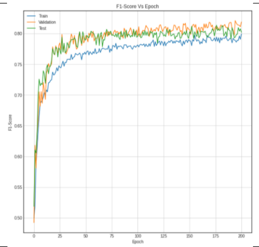	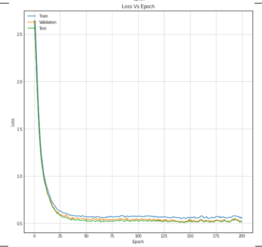	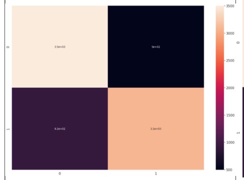	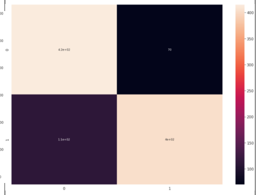	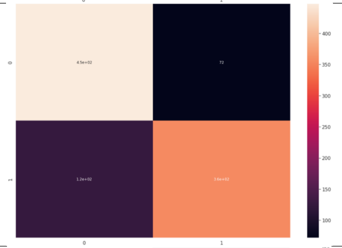
4 [13]	3	200 [14]	0,7637	0,5577	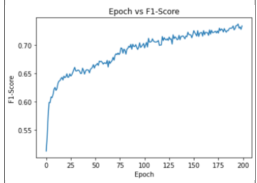	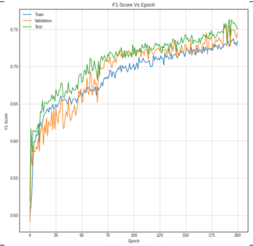	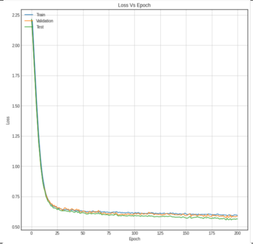	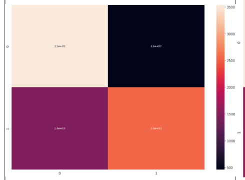	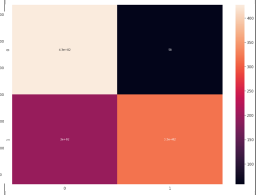	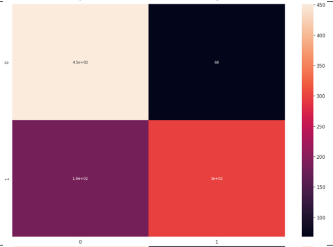
3	3	200	0,832	0,4815	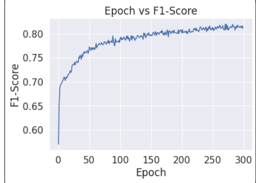	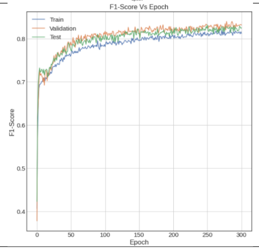	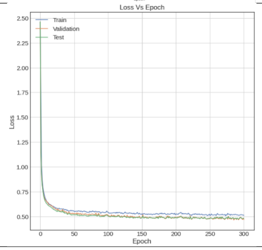	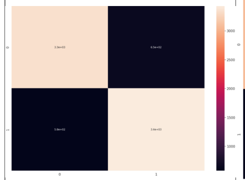	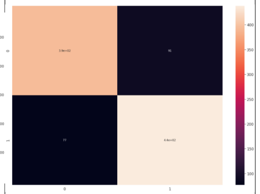	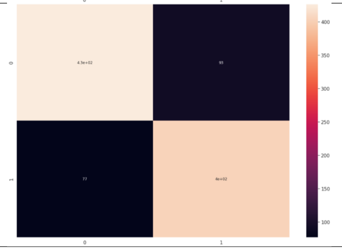

CNN con una muestra de 30.000 secuencias										
**Convoluciones**	**Capas**	**Neuronas**	**F1-score**	**Loss**	**Gráfica F1-score vs épocas**	**Gráfica F1-score vs épocas**	**Gráfica pérdida vs épocas**	**Matriz de confusión Train**	**Matriz de confusión Validation**	**Matriz de confusión Test**
3 [15]	3	200 [16]	0,8805	0,3364	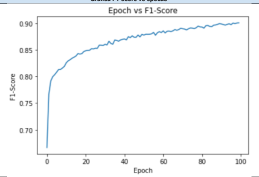	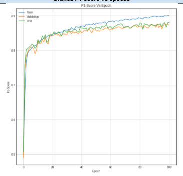	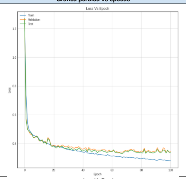	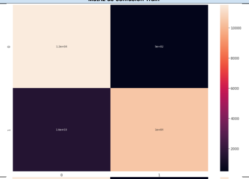	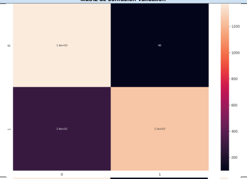	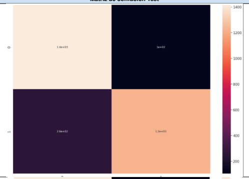
5 [17]	3	200	0,8813	0,4084	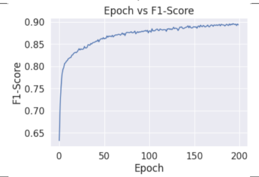	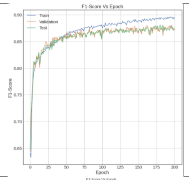	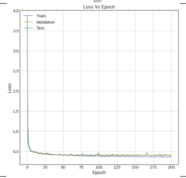	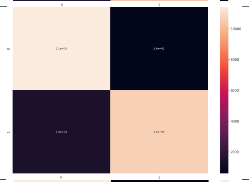	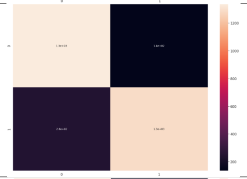	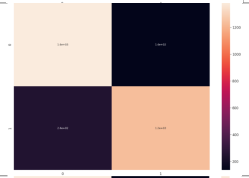
5 [18]	5	200	0,857	0,5929	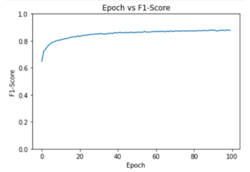	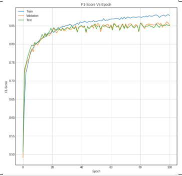	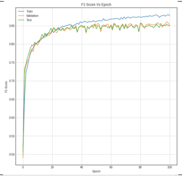	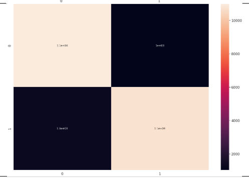	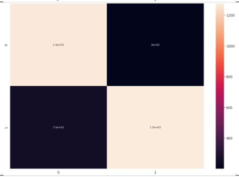	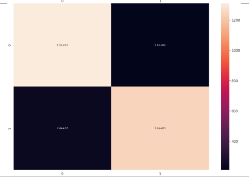

[1] Primera prueba
Dropout = 0.5 en todas las capas, excepto en la primera de FNN con 0.2
Regularización = 0

[2] Misma prueba con 200 epocas
Dropout = 0.5
Lasso {kernel regulation = 0.0001, Bias regulation = 0.01}

[3] Leakyrelu como función de activación (alpha = 0.1)

[4] Misma prueba con 200 epocas
Dropout = 0.5
Lasso {kernel regulation = 0.0001, Bias regulation = 0.01}

[5] Ativation = relu

[6] Misma prueba con 400 epocas
Dropout = 0.5
Lasso {kernel regulation = 0.0001, Bias regulation = 0.01}

[7] Dropout = 0.5
Lasso {kernel regulation = 0.0001, Bias regulation = 0.01}
Activation = LeakyRelU (alpha = 0.1)

[8] Misma prueba con 200 epocas
Dropout = 0.5
Lasso {kernel regulation = 0.00001, Bias regulation = 0.01}

[9] Activation = Leakyrelu

[10] Misma prueba con 400 epocas
Dropout = 0.5
Lasso {kernel regulation = 0.0001, Bias regulation = 0.01}

[11] l1 = 0.0001
kernel_size = (3,3) y (1,3)
filtros = 32, 64, 128
Batchnormalizatión()

Dropout = 0.5

[12] l1 = 0.0001
bias = 0.01
batchnormalization(momentum = 0.5)
Dropout = 0.5

[13] l1 = 0.0001
kernel_size = (3,3) y (1,3)
filtros = 32, 64, 128, 256
Batchnormalizatión()
Dropout = 0.5

[14] l1 = 0.0001
bias = 0.01
batchnormalization(momentum = 0.5)
Dropout = 0.5

[15] Usando convolucionales de: 64,32, 32 respectivamente

[16] Con dropout de 0.2

[17] Usando convoluciones de 32, 64, 128, 128, 128

[18] Usando convolucionales de: 16,32,64,64,128 respectivamente
