# Supplementary material for: Automatic curation of LTR retrotransposon libraries from plant genomes through machine learning
Source: J Integr Bioinform. 2022 Jul 12;19(3):20210036. doi: 10.1515/jib-2021-0036 (PMC9521825; doi:10.1515/jib-2021-0036)
Supplement: Supplementary file 5 — Supplementary Material Details [file j_jib-2021-0036_suppl_005.pdf]

Software	Species	Execution time (seconds)	Total number of sequences	Number of filtered sequences	TN (True Negative)	FN (False Negative)	FP (False Positive)	TP (True Positive)	Precision	Recall	Specificity/ False positive rate	F1-score	Accuracy	Confusion Matrix
LTR_STRUC	***Oryza indica***	3,73	854	404	348	102	29	372	0,927	0,784	0,734	0,85	0,846	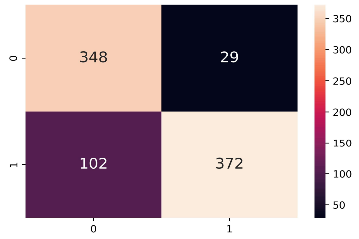
	Oryza granulata	22,61	5734	3148	2302	272	132	2994	0,956	0,916	0,704	0,936	0,929	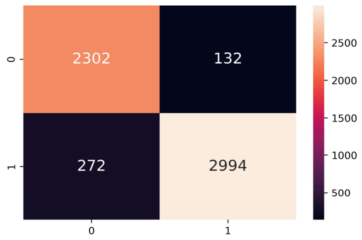
	Coffea eugenioides	20,59	3590	2263	1039	280	96	2156	0,957	0,885	0,4265	0,919	0,894	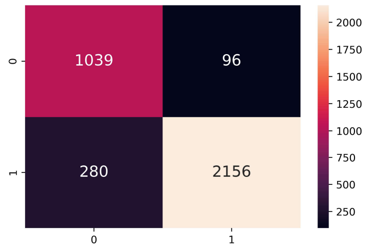
	Coffea humblotiana	8,5	2533	1474	799	260	100	1370	0,931	0,84	0,49	0,883	0,857	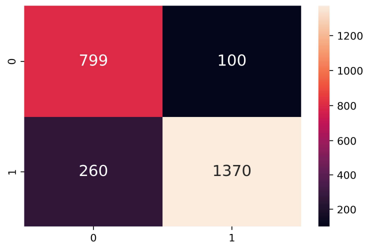

LTR_FINDER	***Oryza indica***	11,84	923	396	444	409	290	424	0,593	0,509	0,533	0,548	0,553	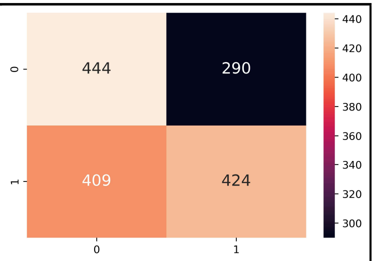
	Oryza granulata	53,01	8597	4700	3752	4138	3328	4730	0,586	0,533	0,423	0,558	0,531	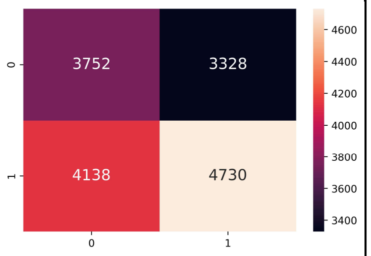
	Coffea eugenioides	29,01	6872	4090	2218	4279	1878	4476	0,704	0,511	0,253	0,592	0,52	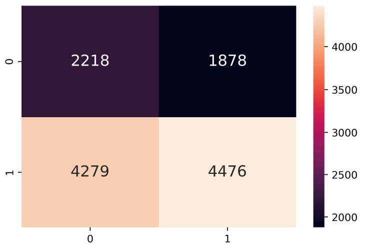
	**Coffea humblotiana**	18,09	2659	1496	932	1623	615	1618	0,724	0,499	0,2875	0,591	0,532	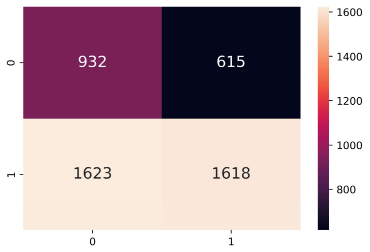
